# Modulation of dendritic cell metabolism by an MPLA-adjuvanted allergen product for specific immunotherapy

**DOI:** 10.3389/fimmu.2022.916491

**Published:** 2022-08-17

**Authors:** Jennifer Zimmermann, Alexandra Goretzki, Clara Meier, Sonja Wolfheimer, Yen-Ju Lin, Hannah Rainer, Maren Krause, Saskia Wedel, Gerd Spies, Frank Führer, Stefan Vieths, Stephan Scheurer, Stefan Schülke

**Affiliations:** ^1^ Vice President´s Research Group 1: Molecular Allergology, Paul-Ehrlich-Institut, Langen, Germany; ^2^ Z6 Occupational Safety, Paul-Ehrlich-Institut, Langen, Germany; ^3^ Division of Allergology, Batch Control and Allergen Analytics, Paul-Ehrlich-Institut, Langen, Germany

**Keywords:** MPLA: monophosphoryl lipid A, immune metabolism, Warburg Effect, allergen specific immunotherapy, vaccine

## Abstract

**Background:**

Recently, bacterial components were shown to enhance immune responses by shifting immune cell metabolism towards glycolysis and lactic acid production, also known as the Warburg Effect. Currently, the effect of allergen products for immunotherapy (AIT) and commercial vaccines on immune cell metabolism is mostly unknown.

**Objective:**

To investigate the effect of AIT products (adjuvanted with either MPLA or Alum) on myeloid dendritic cell (mDC) metabolism and activation.

**Methods:**

Bone marrow-derived mDCs were stimulated with five allergoid-based AIT products (one adjuvanted with MPLA, four adjuvanted with Alum) and two MPLA-adjuvanted vaccines and analyzed for their metabolic activation, expression of cell surface markers, and cytokine secretion by ELISA. mDCs were pre-incubated with either immunological or metabolic inhibitors or cultured in glucose- or glutamine-free culture media and subsequently stimulated with the MPLA-containing AIT product (AIT product 1). mDCs were co-cultured with allergen-specific CD4+ T cells to investigate the contribution of metabolic pathways to the T cell priming capacity of mDCs stimulated with AIT product 1.

**Results:**

Both the MPLA-containing AIT product 1 and commercial vaccines, but not the Alum-adjuvanted AIT products, activated Warburg metabolism and TNF-α secretion in mDCs. Further experiments focused on AIT product 1. Metabolic analysis showed that AIT product 1 increased glycolytic activity while also inducing the secretion of IL-1β, IL-10, IL-12, and TNF-α. Both rapamycin (mTOR-inhibitor) and SP600125 (SAP/JNK MAPK-inhibitor) dose-dependently suppressed the AIT product 1-induced Warburg Effect, glucose consumption, IL-10-, and TNF-α secretion. Moreover, both glucose- and glutamine deficiency suppressed secretion of all investigated cytokines (IL-1β, IL-10, and TNF-α). Glucose metabolism in mDCs was also critical for the (Th1-biased) T cell priming capacity of AIT product 1-stimulated mDCs, as inhibition of mTOR signaling abrogated their ability to induce Th1-responses.

**Conclusion:**

The AIT product and commercial vaccines containing the adjuvant MPLA were shown to modulate the induction of immune responses by changing the metabolic state of mDCs. Better understanding the mechanisms underlying the interactions between cell metabolism and immune responses will allow us to further improve vaccine development and AIT.

## Graphical Abstract

Stimulation of myeloid dendritic cells (mDCs) with an MPLA-adjuvanted, allergoid-based product for allergen immunotherapy (AIT) resulted in increased glycolytic activity while also inducing secretion of the cytokines IL-1b, IL-10, IL-12, and TNF-a. Here, mTOR-dependent glycolysis was shown to regulate the Warburg Effect, glucose consumption, as well as IL-10- and TNF-a secretion induced by the AIT product. While glutamine metabolism also contributed to the activation of mDC metabolism and cytokine secretion, fatty acid metabolism was found to be dispensable for mDC activation. In addition, the glucose metabolism of mDCs was critical for the (Th1-biased) T cell priming capacity of the AIT product-stimulated mDCs, as inhibition of mTOR signaling abrogated their ability to induce Th1-responses. AIT, allergen immunotherapy; mDCs, myeloid dendritic cells; mTOR, mammalian target of rapamycin; MPLA, monophosphoryl lipid A; OXPHOS, oxidative phosphorylation; AAM, amino acid metabolism; FAS, fatty acid synthase; FAO, fatty acid oxidation; 2-DG, 2-deoxyglucose; BPTES, bis-2-(5-phenylacetamido- 1,3,4-thiadiazol-2-yl) ethyl sulfide; TC, T cell.

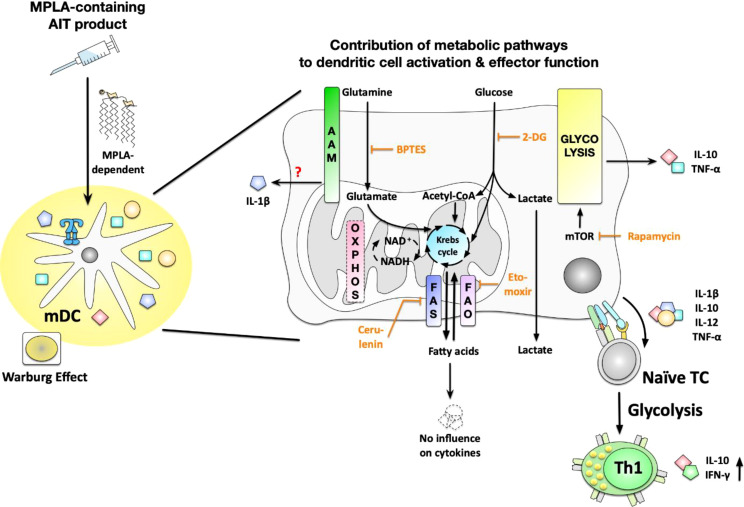

## Introduction

The introduction of vaccines that protect billions of people from preventable diseases can be considered the greatest success of modern medicine. However, both developing novel vaccines and better understanding the mechanisms by which existing vaccines activate innate immune cells to trigger subsequent antigen-specific immune responses are still very important tasks.

We previously reported a fusion protein consisting of the TLR5-ligand flagellin and the major birch pollen allergen Bet v 1 (rFlaA:Betv1) to activate the metabolism of both myeloid dendritic cells (mDCs) (1) and macrophages (2). Here, activation of immune cell metabolism by rFlaA:Betv1 was intricately connected to immune cell effector function as inhibition of immune cell metabolism selectively impaired their capacity to produce certain cytokines while leaving other cytokines unaltered (1, 2).

Indeed, recent high-impact publications have shown activated immune cells to undergo distinct metabolic changes, which not only fulfill the energy needs of these cells but also contribute to their effector function (reviewed in (3)). For example, innate immune cells like DCs and macrophages stimulated by TLR-ligands or pathogens can undergo a switch towards Warburg metabolism (4). In this process, the cells predominantly produce lactate from glucose instead of completely oxidizing glucose to CO2 in their mitochondria (4). Warburg metabolism, also called aerobic fermentation, is both an oxygen-independent (acutely inflamed tissues are often highly hypoxic) and faster way of producing energy. Warburg metabolism is energetically less efficient as it only generates two molecules of ATP per molecule glucose, whereas complete oxidation of glucose *via* the mitochondria results in the formation of 36 molecules of ATP per molecule glucose. However, activated innate immune cells have elegantly adapted to their need to function under conditions of low oxygen supply in inflamed tissues by using a “disrupted” Krebs cycle (resulting from the undersupply of mitochondria during Warburg metabolism) for the generation of important immune effector molecules such as reactive oxygen species (ROS), reactive nitrogen species (NOS), prostaglandins, or itaconate (5). These results have led to the formation of the new research field “immune metabolism”.

While the effects of TLR-ligands on immune cell metabolism are now much better understood, the effect of commercially available vaccine preparations or products for allergen immunotherapy (AIT) on immune cell metabolism is currently unknown.

Pure protein antigens used for vaccination generally have a low immunogenicity (6). Therefore, in addition to the antigen, vaccines usually contain adjuvants to enhance immune responses (7). Adjuvants are substances such as amino acids (L-Tyrosine), inorganic aluminum salts (Alum), or pathogen-associated molecular patterns (PAMPs) (such as monophosphoryl lipid A (MPLA)) that by activating certain immune cells, enhance the immunogenicity of the respective antigen (8, 9).

MPLA, which has become widely used as an adjuvant in clinical practice, is a derivative of the lipopolysaccharide (LPS) of gram-negative bacteria (10, 11). It activates TLR4 but with lower toxicity than LPS (10, 12, 13). MPLA is used in a variety of commercial vaccines e.g. against rabies (14), hepatitis B, and human papillomavirus (15). It has been shown that MPLA is not only unable to activate murine and human mast cells *in vitro (* 13) but furthermore promotes Th1 responses in mice (16). This combination of reduced mast cell activation and the shift from Th2- to Th1 responses makes MPLA an interesting adjuvant for treating allergic diseases (17).

We recently showed MPLA to mTOR- and JNK-MAPK-dependently activate glucose metabolism in mDCs characterized by an induction of the Warburg Effect, increased glucose consumption from the culture medium, as well as pro- and anti-inflammatory cytokine secretion (18). After describing the immune-metabolic effects of MPLA on mDCs (18), in this study we investigated the effect of MPLA in combination with allergens to evaluate if MPLA-containing AIT products also have immune-metabolic effects.

There is one MPLA-containing AIT product Pollinex^®^ Quattro (from here on termed AIT product 1), that is currently being investigated in clinical trials for the treatment of allergies, particularly hay fever (19). It contains pollen allergens derived from birch, alder, and hazel that are modified with glutaraldehyde (19). In addition, the mixture contains MPLA as an adjuvant to enhance the immune response and L-Tyrosine, which is used as a depot to ensure a steady release of antigens, thus preventing severe inflammatory reactions (17). Currently, the activation of dendritic cells by AIT product 1 is under-investigated, and the immune-metabolic effects of AIT product 1 are unknown.

In the present study, we (I) compared AIT product 1 to four other allergoid-based AIT products adjuvanted with Alum and two commercial vaccine preparations adjuvanted with MPLA and (II) investigated the immune-metabolic activation induced by AIT product 1 and its underlying mechanisms.

In summary, we found the AIT product 1 and the two tested commercial vaccines (all containing the adjuvant MPLA) to modulate the induction of immune responses by changing the metabolic state of dendritic cells. Better understanding the mechanisms underlying the interactions between immune cell metabolism and immune responses will allow us to improve vaccine development and AIT.

## Material and methods

### Mice

C57BL/6J and BALB/c mice were obtained from Jackson Laboratories, Maine, USA, and maintained under specific pathogen-free conditions at the Paul-Ehrlich-Institut in Langen, Germany. All animal experiments were performed in compliance with the German animal protection law (animal license approval number F107/1049).

### *In vitro* generation of mouse bone marrow-derived dendritic cells, stimulation, and ELISA analysis

Mouse mDCs were generated as described previously (20). Briefly, for the generation of myeloid DCs (mDCs) bone marrow cells were cultured in complete culture medium (RPMI1640, Gibco, Karlsruhe, Germany supplemented with 10% FCS, 1 mM sodium pyruvate, 10 mM HEPES, penicillin (100 U/ml), streptomycin (100 µg/ml), and 0.1 mM 2-mercaptoethanol) supplemented with 100 ng/ml recombinant mouse GM-CSF for 8 days and medium was changed every 2 days. For generation of plasmacytoid DCs (pDCs) bone marrow cells were cultured in complete culture medium supplemented with 100 ng/ml recombinant mouse Flt-3L (R&D, Abingdon, UK) for 8 days and medium was changed once on day 4. On day 8, DCs were harvested as the loosely adherent cell population without scratching of the strongly adherent macrophages, seeded at a concentration of 5x105 cells/ml in 24-well plates (ThermoScientific, Dreieich, Germany) and stimulated with either the indicated AIT products or vaccines for 72 h. LPS (10 µg/ml, L5886, Sigma Aldrich, Taufkirchen, Germany) served as a positive control. Bone marrow-derived mDCs were pre-incubated in either complete culture medium, glucose-free, or glutamine-free medium for 90 min prior to stimulation for additional 72 h. Supernatants were analyzed for cytokine secretion by ELISA according to (2).

### Reagents

Commercially available preparations of all AIT products and vaccines were used. For the investigation of different allergen-containing AIT products, an MPLA-containing allergoid-based AIT product (Pollinex^®^ Quattro, Bencard Allergy, Munich, Germany, termed AIT product 1) was compared to four other allergoid-based, Alum-adjuvanted AIT products: ALLERGOVIT^®^ BEH (Allergopharma, Reinbek, Germany, named AIT product 2), Purethal^®^ Spring trees I (HAL Allergy, Leiden, The Netherlands, named AIT product 3), ALLERGOVIT^®^ Birch (Allergopharma, named AIT product 4), Purethal^®^ Birch (HAL Allergy, named AIT product 5), as well as two other MPLA-containing vaccine preparations: Fendrix^®^ for the prevention of hepatitis B (GlaxoSmithKline, Brentford, UK, named vaccine 1) and Cervarix^®^ for the prevention of cervical cancer (GlaxoSmithKline, named vaccine 2). See **Table 1** for further information on the coding, indications, and composition of the respective products.

**Table 1:** Coding, indications, and composition of the used AIT products and vaccines.

Code name	Product name	Vendor	Indication	Contained Antigen(s)	Adjuvant
AIT product 1	Pollinex^®^ Quattro BEH	Bencard, Allergy	Allergy treatment	300, 800, or 2000 standardized units (SU)/ml tree pollen allergoids (birch, elder, hazel) adsorbed to L-Tyrosine	50 µg MPLA per 1.5 ml syringe
AIT product 2	ALLERGOVIT^®^ BEH	Allergopharma	Allergy treatment	10000 therapeutic units (TE)/ml tree pollen allergoids (35% birch, 30% elder, 35% hazel)	Aluminum hydroxide
AIT product 3	Purethal^®^ Spring trees I	HAL Allergy	Allergy treatment	20000 arbitrary units (AUM)/ml tree pollen allergoids (33% birch, 33% elder, 33% hazel)	0.55 mg aluminum hydroxide per 3 ml solution
AIT product 4	Allergovit^®^ birch	Allergopharma	Allergy treatment	10000 TE/ml birch pollen allergoids	Aluminum hydroxide
AIT product 5	Purethal^®^ birch	HAL Allergy	Allergy treatment	20000 AUM/ml birch pollen allergoids	0.55 mg aluminum hydroxide per 3 ml solution
Vaccine 1	Fendrix^®^	GlaxoSmithKline	Prevention of hepatitis B infection	20 µg Hepatitis B surface antigen per 0.5 ml syringe	AS04 (50 µg MPLA & adsorbed to 0.5 mg aluminum phosphate per 0.5 ml syringe)
Vaccine 2	Cervarix^®^	GlaxoSmithKline	Prevention of cervical cancer	40 µg recombinant human papilloma virus L1 proteins per 0.5 ml syringe	AS04 (50 µg MPLA & adsorbed to 0.5 mg aluminum phosphate per 0.5 ml syringe)

BEH, birch; elder, hazel; SU, standardized units; TE, therapeutic units; AUM, arbitrary units; MPLA, monophosphoryl lipid A; AS, adjuvant system.

The protein content was determined according to Ph. Eur. monograph 2.5.33 (method 7), using Kjeldahl´s method followed by Berthelot reaction. First the samples were washed according to the manufacturer’s informations in order to remove L-Tyrosine, followed by protein precipitation using tungstophosphoric acid. Protein digestion was performed at 37°C using concentrated sulphuric acid and Kjeldahl´s catalyst. The resulting ammonia was quantitatively determined by Continuous Flow Analysis using the Berthelot´s reaction. Protein concentrations were calculated against a BSA standard in protein nitrogen units (PNU/ml). Total protein concentrations of the used AIT products are listed in **Table 2**.

**Table 2:** Total protein concentrations of the used AIT products as determined by Kjeldahl measurement.

AIT product	Allergen concentration provided by manufacturer	Total protein concentration in PNU/ml
**AIT product 1**	2000 standardized units (SU)/ml	319
**AIT product 2**	10000 therapeutic units (TE)/ml	4493
**AIT product 3**	20000 arbitrary units (AUM)/ml	2888
**AIT product 4**	10000 TE/ml	3429
**AIT product 5**	20000 AUM/ml	2878

SU, standardized units; TE, therapeutic units; AUM, arbitrary units.

For control purposes MPLA was acquired from *In vivo*Gen (Toulouse France) and L-Tyrosine from Sigma-Aldrich. For stimulations of mDCs with either MPLA, L-Tyrosine, MPLA + L-Tyrosine, or AIT product 1 the amounts of MPLA and L-Tyrosine were adjusted to reflect the amounts of MPLA and L-Tyrosine contained within the applied concentrations of AIT product 1, respectively.

### Inhibitor experiments

For inhibition experiments, mDCs were pre-incubated with the indicated amounts of either rapamycin (mTOR inhibitor), SP600125 (SAP/JNK MAPK inhibitor, both *In vivo*gen, Toulouse, France), BPTES (glutaminase inhibitor), cerulenin (inhibitor of fatty acid synthase), etomoxir (inhibitor of carnitine palmitoyltransferase 1, all Sigma-Aldrich), or 2-deoxyglucose (2-DG, hexokinase 2 inhibitor, Carl-Roth Laboratory Supplies, Karlsruhe, Germany) for 90 min and subsequently stimulated with AIT product 1 (containing 2000 SU/ml of allergens if not specified otherwise) for 72 h. The toxicity of the used inhibitors was determined using the fixable viability dye eFlour780 (ThermoFisher Scientific, data not shown). Inhibitor concentrations showing toxic effects were excluded from the analysis.

### Analysis of cell metabolic state

The Warburg Effect in stimulated mDC cultures was determined photometrically 72 h post-stimulation by quantifying the OD at 570 nm and calculated as 1/OD(570nm) normalized to unstimulated controls. Glucose concentrations in culture supernatants were determined 72 h post-stimulation using the Glucose (GO) Assay Kit (Sigma-Aldrich). The metabolic rate was derived from the measured glucose concentrations by calculating the glucose consumption in % in comparison to medium without mDCs (glucose concentration in RPMI1640 = 2 mg/ml).

### Extracellular flux assays

For metabolic flux analysis, 1x105 mDCs per well were seeded in Seahorse XF96 cell culture microplates (Agilent Technologies, Santa Clara, CA, USA) for one hour at RT and subsequently transferred to a CO2 incubator for overnight incubation at 37°C, 5% CO2, and 80% humidity. On the next day, the mDC medium was exchanged to Seahorse XF assay medium and the Seahorse XF Test was performed according to the manufacturer’s recommendations using a Seahorse XFe96 Analyzer from Agilent Technologies. Cycle numbers were as follows: Initially, four cycles of baseline measurement were performed to obtain a stable signal. Afterwards, cells underwent either (I) 14 cycles of stimulation with either the indicated vaccines or AIT product 1 with or without pre-treatment of 50 mM 2-DG for eight cycles or (II) 14 cycles of stimulation with AIT product 1 or the two MPLA-containing vaccines followed by 8 cycles with either oligomycin-, rotenone/antimycin A- (Rot/AA), and 2-DG-treatment, respectively (1 cycle = 3 min mixing plus 3 min measuring). Cells were continuously analyzed for extracellular acidification rates (ECAR) and oxygen consumption rates (OCR). Upon completion of the measurement, samples were centrifuged, lysed, and normalized to total protein amount *via* BCA (ThermoFisher Scientific, Darmstadt, Germany), analyzed using the Wave software from Agilent Technologies, and visualized using Graphpad Prism (version 9 for MacOS and Windows, GraphPad Software, Inc., San Diego, CA; USA). Percentages given in the graphs for ECAR and OCR values refer to normalized % increases compared to the last baseline cycle before cells were stimulated with the indicated stimuli.

### Flow cytometry

The activation of mDCs was assessed by flow cytometry using anti-mouse PE-conjugated CD40 (clone: 1C10, dilution: 1 to 100), PE-conjugated CD69 (clone: H1.2F3, dilution: 1 to 150), FITC-conjugated CD80 (clone: 16-10A1, dilution: 1 to 50), FITC-conjugated CD86 (clone: GL1, dilution: 1 to 50, all eBiosciences, Frankfurt, Germany), as well as respective isotype controls. Additionally, cells were stained with anti-mouse pacific blue-conjugated CD11b (clone: M1/70.15, dilution: 1 to 50, Invitrogen, ThermoFisher Scientific), allophycocyanin-conjugated CD11c (clone: HL3, dilution: 1 to 500, BD Bioscience), and PE-Cy5-conjugated B220 (clone: RA3-6B2, dilution: 1 to 100, BD Bioscience) with their respective isotype controls. FITC or PE intensity of CD11b+CD11c+B220− (mDC) cells was quantified by FACS using a FORTESSA flow cytometer (BD Bioscience). Data were analyzed using FlowJo V.7 (Treestar Inc., Ashland, OR, USA).

### mDC and T cell co-cultures

mDCs isolated and differentiated from BALB/c mice were seeded into 48-well plates with a seeding concentration of 1.6x105 cells per well in a total volume of 500 µl. The mDCs were treated with the indicated concentrations of the different metabolic inhibitors for 16 hours. After the 16 h incubation, plates were washed by centrifugation at 1200 rpm (226 g) for 5 minutes at 4°C. The media were carefully removed by pipetting and 250 µl of fresh 37°C complete culture medium was added. Subsequently, T cells isolated from the spleens of Bet v 1/Alum-immunized BALB/c mice (two times 10 µg rBet v 1 plus 10 mg Alum in a total volume of 200 µl PBS i.p., two weeks apart (1)) were added to the mDCs in a seeding concentration of 6.3x105 cells in a volume of 125 µl. Subsequently, the co-cultures were stimulated with AIT product 1, and re-stimulated with rBet v 1 (21), and the total culture volume was adjusted to 500 µl. Co-cultures were incubated at 37°C, 5% CO2, 80% humidity for 72 h, and supernatants were collected and frozen at -20°C until measurement.

### Statistical analyses

For statistical analyses, the data sets were first checked for gaussian normal distribution. For normally distributed data sets a ONE-way ANOVA with correction for multiple comparisons according to Tukey was used to compare independent experiments. For non-normally-distributed data sets, a Kruskal-Wallis test with correction for multiple comparisons according to Dunn was used. All statistical evaluations were performed using GraphPad Prism software Version 9. Statistical significance was achieved at p<0.05 with the following specifications: * = p < 0.05, ** = p < 0.01, *** = p < 0.001, n.s. = non-significant result.

## Results

### Both the MPLA-containing allergen product for AIT and MPLA-containing vaccines activate mDC metabolism

**Figure 1:**
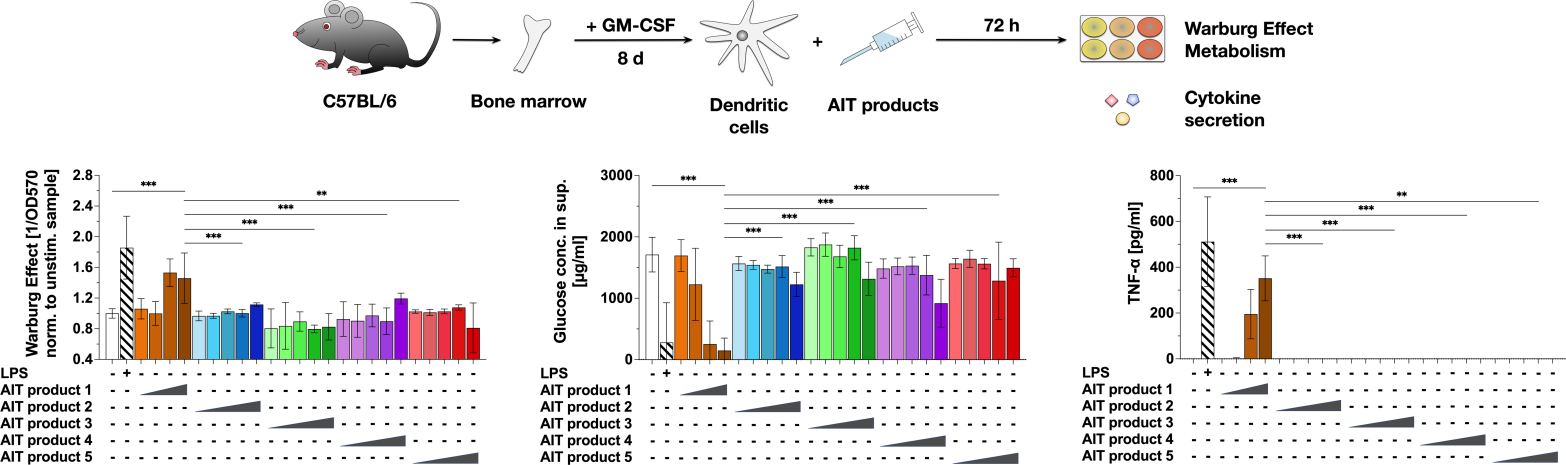
An MPLA-containing AIT product can activate the metabolism of dendritic cells. C57BL/6 bone marrow-derived mDCs were stimulated with either 0.025, 0.25, 2.5, 5, or 50 (only for AIT products 2 to 5) PNU/ml of the different AIT products (see **Table 1** for coding and composition of the products and **Table 2** for protein concentrations) or 10 µg/ml LPS as a positive control for 72 h and analyzed for the activation of mDC metabolism and cytokine secretion. The Warburg Effect, glucose consumption from the culture medium, and TNF-α secretion were determined 72 h post-stimulation. Data are mean results of three independent experiments ± SD. Data displayed no gaussian normal distribution. For statistical analysis a Kruskal-Wallis test with correction for multiple comparisons according to Dunn was applied. Statistical significance was achieved at **p<0.01, ***p<0.001, respectively with “n.s.” representing non-significant results.

After showing that MPLA can activate glucose metabolism in mDCs (18), we investigated the immune-metabolic effects of allergen preparations currently used for AIT that are adjuvanted with either MPLA or Alum. Therefore, we compared one commercially available, MPLA-containing, allergoid-based AIT product (AIT product 1) with four other Alum-adjuvanted, allergoid-based AIT products (AIT products 2 to 5, see **Tables 1**, **2** for more information on the respective products and **Figure 1**).

For this, mDCs were differentiated from mouse bone marrow, stimulated with the same total protein amounts (normalized to PNU/ml determined by Kjeldahl) of the different AIT products for 72 h, and analyzed for the activation of mDC metabolism and cytokine secretion (**Figure 1**). In comparison to the Alum-containing AIT products 2 to 5, only the MPLA-containing AIT product 1 dose-dependently induced a Warburg Effect, an increase in glucose consumption from the culture medium, and secretion of the pro-inflammatory cytokine TNF-α (**Figure 1**). Even applying 10-fold higher total protein amounts of AIT products 2 to 5 (5 PNU/ml maximum stimulation concentration of AIT product 1 vs. 50 PNU/ml for AIT products 2 to 5) did not result in either metabolic activation or cytokine secretion (**Figure 1**). Similar results were obtained for mDCs differentiated from BALB/c mice (**Repository Figure E1**), while no activation of metabolism and TNF-α secretion was detected upon stimulation of Flt-3L cultures (**Repository Figure E2**).

**Figure 2:**
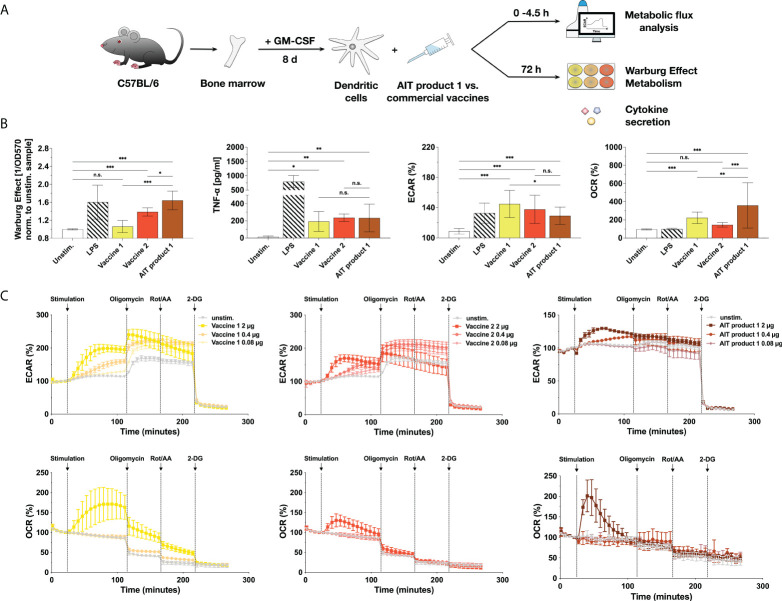
MPLA-containing vaccine preparations also activate the metabolism of dendritic cells. C57BL/6 bone marrow-derived mDCs were stimulated with either AIT product 1, two commercial, MPLA-containing vaccines (see **Table 1** for coding and composition of the products), or 10 µg/ml LPS as a positive control for 72 h and analyzed for the activation of mDC metabolism and cytokine secretion **(A)**. Cells were analyzed for the induced Warburg Effect and TNF-α secretion 72 h post-stimulation and extracellular acidification rates (ECAR) and oxygen consumption rates (OCR) in cycle 7 (42 min) post-stimulation using Seahorse technology **(B)**. Stimulation concentrations for all three tested products were normalized to contain 0.76 µg/ml of MPLA (corresponding to a total protein amount of 5 PNU/ml of AIT product 1). For Extracellular Flux Assays, mDCs adhered to the plastic plate were stimulated with either 100 ng/ml of LPS or the indicated stimulation concentrations of AIT product 1 or vaccine 1 and 2 (with stimulation concentrations normalized to their MPLA content) for the indicated durations (B,C). 14 cycles (84 min) post-stimulation, the ATP synthase, the electron transfer chain, and glycolysis were inhibited, respectively, by sequential injection of oligomycin, rotenone/antimycin A (Rot/AA), and 2-deoxy-glucose (2-DG) for 8 cycles (48 min) each. Data are either representative **(C)** or mean results **(B)** of three to five independent experiments ± SD Data displayed a gaussian normal distribution. For statistical analysis a ONE-way ANOVA with correction for multiple comparisons according to Tukey was applied. Statistical significance was achieved at *p<0.05, **p<0.01, ***p<0.001, respectively with “n.s.” representing non-significant results.

To investigate whether the immune-metabolic properties of AIT product 1 are also observed for other MPLA-containing products, AIT product 1 was directly compared to two other commercial vaccine preparations that employ MPLA as an adjuvant (vaccine 1 and 2, see **Table 1** for coding and exact composition of the vaccines, **Figure 2A**).

When comparing multiple independent experiments, AIT product 1 induced the strongest Warburg Effect (1.06-fold higher compared to unstimulated controls for vaccine 1, 1.39 for vaccine 2, and 1.64 for AIT product 1), while all three products induced identical levels of TNF-α secretion (**Figure 2B**).

The activation of mDC metabolism by the three MPLA-containing products was further investigated using Seahorse Extracellular Flux Assays (**Figures 2B**, **C**). Interestingly, all investigated products led to comparable increases in extracellular acidification rates (ECAR, 145% for vaccine 1, 138% for vaccine 2, and 129% for AIT product 1 compared to 109% obtained for unstimulated controls) (corresponding to an increased generation of lactate from glucose) while also boosting rates of oxygen consumption (OCR) to varying degrees (223% for vaccine 1, 145% for vaccine 2, and 358% for AIT product 1 compared to 95% for unstimulated controls) (**Figure 2B**).

A more in-depth analysis of mDC metabolism upon stimulation with AIT product 1 and both vaccines showed all three stimuli to result in a dose-dependent increase in glycolysis (increased ECAR values) which was sustained throughout the whole measurement (**Figure 2C**). Moreover, stimulation with all three products also resulted in a burst in oxygen consumption (increased OCR values, **Figure 2C**). This burst in OCR was more pronounced for vaccine 1 and AIT product 1 compared to vaccine 2 (**Figure 2C**). Inhibition of mitochondrial respiration by sequential injection of oligomycin and rotenone/antimycin A resulted in a step-wise reduction in OCR, paralleled by a compensatory increase in ECAR for vaccines 1 and 2 but not AIT product 1 (**Figure 2C**). Finally, inhibition of glucose metabolism by 2-deoxyglucose (2-DG) completely suppressed cellular metabolism (**Figure 2C**).

Therefore, our results suggested that certain commercial vaccines and one AIT product containing the adjuvant MPLA can activate mDC metabolism characterized by both increased rates of glycolysis and a burst in oxygen consumption.

### AIT products and MPLA-adjuvanted vaccines upregulate surface markers expression of bone marrow-derived mDCs

**Figure 3:**
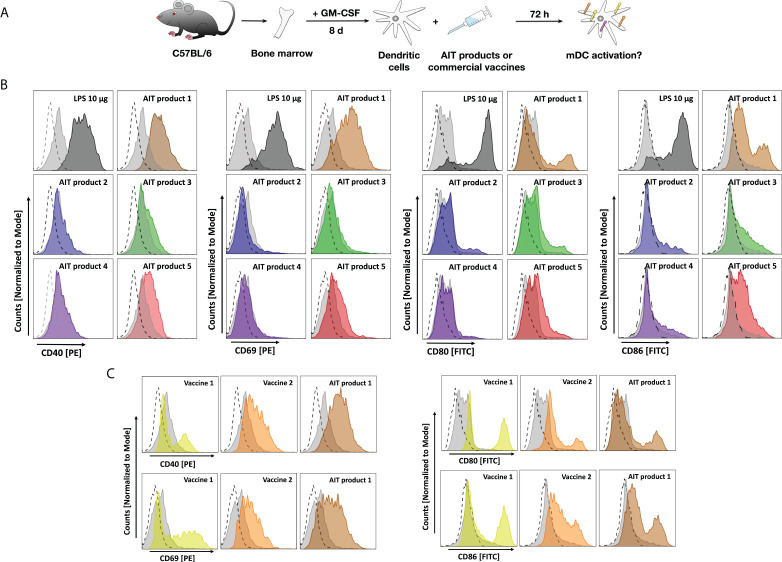
AIT products and MPLA-adjuvanted vaccines upregulate surface marker expression of bone marrow-derived mDCs. C57BL/6 mDCs were stimulated for 3 days with either the indicated AIT products or MPLA-containing vaccines (all corresponding to a total protein amount of 5 PNU/ml of AIT product 1), cells were harvested, and stained for surface expression of CD40, CD69, CD80, or CD86 **(A)**. Expression levels of the indicated maturation and activation markers on CD11b+CD11c+B220- mDCs after stimulation with either AIT products **(B)** or MPLA-containing vaccines **(C)** were determined by flow cytometry. Dashed lines: isotype control-stained cells, light grey filled curves: unstimulated cells, colored curves: stimulated as indicated. Data are representative results taken from one out of two experiments.

To further characterize the activation of marrow-derived mDCs by the different AIT products and vaccines, cells were stimulated with the different stimuli and analyzed for the expression of surface activation and maturation markers by flow cytometry (**Figure 3A**).

AIT product 1 increased expression levels of all investigated surface markers, which were for CD40 and CD69 comparable to the levels observed for the positive control LPS (**Figure 3B**). Furthermore, AIT product 5 also increased expression levels of all investigated markers, albeit to a lower extent compared to AIT product 1 (**Figure 3B**). For AIT products 2 and 4, only a very slight upregulation of CD86 was observed, while AIT product 3 slightly increased expression levels of CD40, CD80, and CD86 (**Figure 3B**). Also, both MPLA-containing vaccines resulted in increased surface expression levels of all investigated markers (**Figure 3C**). Here, AIT product 1 was shown to be the strongest stimulus, followed by vaccine 2 and vaccine 1 (**Figure 3C**).

### MPLA contained in AIT product 1 mediates mDC activation

Subsequently, the immune-metabolic effects of AIT product 1 were further characterized to understand the underlying mechanisms.

Stimulation of mDCs with AIT product 1 induced a dose-dependent activation of glucose metabolism characterized by a significantly increased Warburg Effect, glucose consumption from the culture medium, and metabolic rate (**Repository Figure E3A**). Moreover, AIT product 1 induced a dose-dependent and, for higher stimulation concentrations, highly significant secretion of the cytokines IL-1ß, IL-10, and TNF-α (**Repository Figure E3A**).

Metabolically, the stimulation of mDCs with AIT product 1 resulted in both an increase in ECAR (max. 25% compared to unstimulated cells) as well as a massive increase in OCR (500% compared to the unstimulated controls, **Repository Figure E3B**). However, both ECAR and OCR decreased over the course of the measurement. Inhibition of glycolysis by pre-treatment of the cells with 2-DG prevented the otherwise observed increase in both ECAR and OCR upon stimulation of the cells with AIT product 1 (**Repository Figure E3B**, yellow vs. brown lines). These results further confirm that mDCs, activated by AIT product 1, exhibit a highly glycolytic metabolism.

To determine which component of AIT product 1 mediates the observed mDC activation, bone marrow-derived mDCs were stimulated with different formulations of AIT product 1 containing different allergen amounts (either 300, 800, or 2000 SU/ml), while the concentration of the included adjuvant components like L-Tyrosine or MPLA (50 µg/1.5 ml syringe) was equal for all undiluted formulations (300, 800, or 2000 SU/ml). 72 hours post-stimulation, activation of glucose metabolism and cytokine secretion were measured to determine the contribution of the different amounts of the allergens to the mDC activation (**Repository Figure E4A**).

All tested AIT product 1 formulations dose-dependently induced an equal Warburg Effect (**Repository Figure E4B**), as well as pro- and anti-inflammatory cytokine secretion (**Repository Figures E4C–E** , no statistically significant differences between the highest concentrations of all tested formulations). These results suggest the observed activation of glucose metabolism and secretion of cytokines from mDCs by AIT product 1 to be independent of the amount of allergen in the preparations.

**Figure 4:**
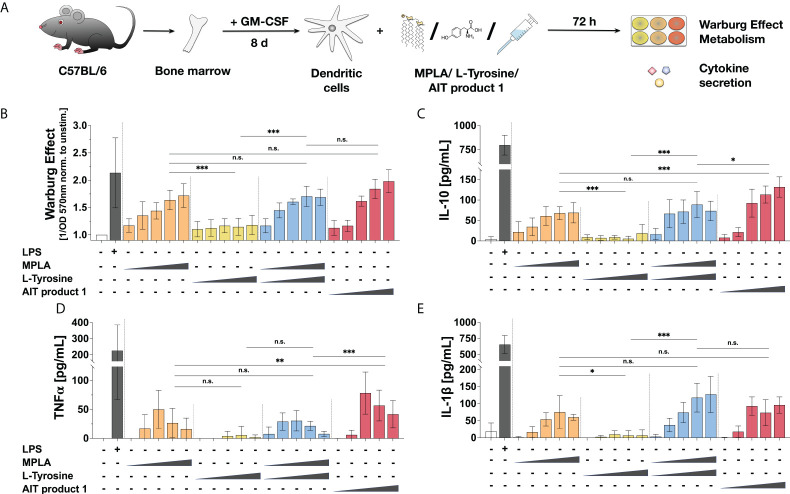
Stimulation of mDCs with MPLA results in similar activation of glucose metabolism and cytokine secretion to AIT product 1. C57BL/6 bone marrow-derived mDCs were stimulated with increasing concentrations of either MPLA, L-Tyrosine, the mixture of MPLA and L-Tyrosine, or AIT product 1 (all equivalent to either 0.025, 0.25, 1.25, 2.5, or 5 PNU/ml of AIT product 1) or 10 µg/ml LPS as a positive control for 72 h and analyzed for the activation of mDC metabolism and cytokine secretion **(A)**. The induced Warburg Effect **(B)** and the secretion of the indicated cytokines **(C–E)** were determined 72 h post-stimulation. Data are mean results of three independent experiments ± SD. Data displayed a gaussian normal distribution. For statistical analysis a ONE-way ANOVA with correction for multiple comparisons according to Tukey was applied. Statistical significance was achieved at *p<0.05, **p<0.01, ***p<0.001, respectively with “n.s.” representing non-significant results.

As AIT product 1 was activating mDCs independently of the amount of allergens contained in different strengths of the product (**Repository Figure E4**), the remaining single adjuvant components contained within the AIT product 1 formulation (MPLA and L-Tyrosine) were investigated for their capacity to activate mDCs (**Figure 4A**). Here, and in all following experiments, the 2000 SU/ml formulation was chosen for availability reasons. We neither observed a change in the Warburg Effect nor cytokine secretion upon treatment with only L-Tyrosine compared to the unstimulated controls (**Figures 4B, C**). A concentration-dependent increase in both the Warburg Effect and IL-10, TNF-α, and IL-1β secretion equal to the stimulation with AIT product 1 was only observed once respective amounts of MPLA were added (**Figures 4B**–**E**). These results suggest, that the MPLA contained in AIT product 1 mainly mediates the observed activation of mDCs.

### AIT product 1-mediated mDC activation is mainly driven by mTOR-dependent glycolysis

**Figure 5:**
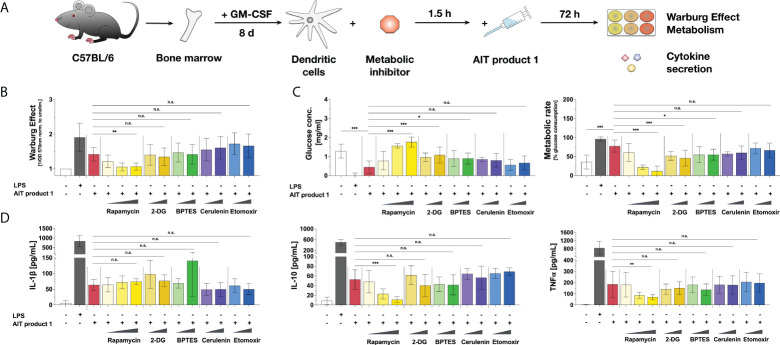
AIT product 1-mediated mDC activation is mainly driven by mTOR-dependent glycolysis. C57BL/6 bone marrow-derived mDCs were pre-treated with the indicated amounts of either the mTOR inhibitor rapamycin (0.1, 1, or 10 nM), the hexokinase 2 inhibitor 2-deoxyglucose (2-DG) (0.01 or 0.1 mM), the amino acid metabolism inhibitor BPTES (0.02 or 0.2 µM), or the inhibitors of fatty acid synthase cerulenin (0.02 or 0.2 µg/ml) and fatty acid oxidation etomoxir (0.05 or 0.5 µM) for 90 minutes and subsequently stimulated with 2.5 PNU/ml AIT product 1 (containing 0.38 µg/ml MPLA) for additional 72 h. 10 µg/ml LPS served as a positive control. mDCs were subsequently analyzed for the activation of mDC metabolism and cytokine secretion **(A)**. The Warburg Effect **(B)**, glucose concentration in the medium and metabolic rates **(C)**, and cytokine secretion **(D)** were determined 72 h post-stimulation. Data are mean results of three independent experiments ± SD. Data displayed a gaussian normal distribution. For statistical analysis a ONE-way ANOVA with correction for multiple comparisons according to Tukey was applied. Statistical significance was achieved at *p<0.05, **p<0.01, ***p<0.001, respectively with “n.s.” representing non-significant results.

To investigate which metabolic pathways contribute to the activation of mDCs by AIT product 1, mDCs were pre-treated with different metabolic inhibitors for 1.5 hours prior to stimulation with AIT product 1. 72 hours post-stimulation, metabolic parameters and cytokine secretion were analyzed (**Figure 5A**). To block glycolysis, 2-DG, which inhibits the glycolytic enzyme hexokinase 2 (22), and rapamycin, which inhibits the mTOR complex 1 (mTORC1), were used (23). For inhibition of amino acid metabolism, the glutaminase inhibitor bis-2-(5-phenylacetamido-1,3,4-thiadiazol-2-yl)-ethyl-sulfide (BPTES) which blocks the conversion of glutamine to glutamate within the mitochondria was employed (24). Finally, inhibition of fatty acid metabolism was achieved through pre-treatment with either cerulenin to block fatty acid synthase (25) or etomoxir to block fatty acid oxidation *via* inhibiting carnitine palmitoyltransferase 1 (26).

The induced Warburg Effect was substantially influenced by the mTORC1-inhibitor rapamycin. Here, mTOR-inhibition dose-dependently suppressed the Warburg Effect to levels observed in the unstimulated control cells (**Figure 5B**). Neither the inhibition of hexokinase 2 by 2-DG nor the inhibition of amino acid metabolism by BPTES or fatty acid metabolism by either cerulenin or etomoxir influenced the AIT product 1-induced Warburg Effect (**Figure 5B**). By contrast, pre-treatment with rapamycin and, to a lower extent (and without dose-dependent effects), 2-DG or BPTES suppressed glucose consumption and metabolic rates, resulting in an increase of glucose levels in mDC supernatants compared to AIT product 1-stimulated controls (**Figure 5C**).

Moreover, mTOR inhibition by pre-treatment with rapamycin resulted in a concentration-dependent decrease in the secretion of IL-10 and TNF-α upon stimulation with AIT product 1 (**Figure 5D**). In contrast, the inhibition of other metabolic pathways like amino acid metabolism (by BPTES), fatty acid metabolism (by either etomoxir or cerulenin), or hexokinase 2 by 2-DG had no effect on cytokine secretion (**Figure 5D**). Interestingly, IL-1β secretion was increased upon inhibition of amino acid metabolism by BPTES, which however did not reach statistical significance (**Figure 5D**) due to intra-experimental variations.

### AIT product 1-mediated mDC activation depends on SAP/JNK MAPK-signaling

**Figure 6:**
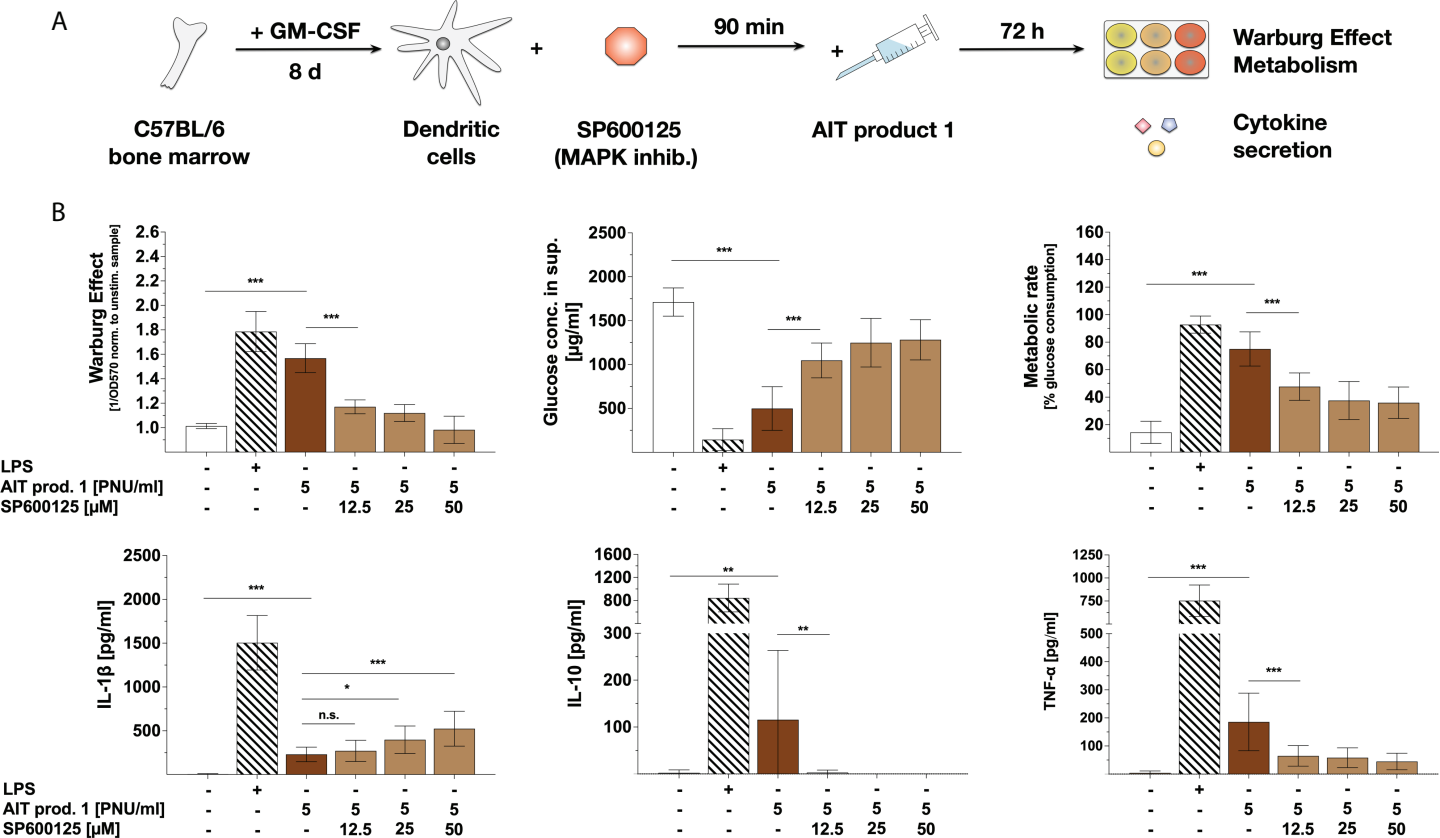
Activation of mDC metabolism, IL-10-, and TNF-α secretion by AIT product 1 depend on SAP/JNK MAPK-signaling. C57BL/6 bone marrow-derived mDCs were pre-treated with the indicated amounts of the SAP/JNK MAPK inhibitor SP600125 for 90 minutes and subsequently stimulated with 5 PNU/ml of AIT product 1 (containing 0.76 µg/ml MPLA) for additional 72 h **(A)**. 10 µg/ml LPS served as a positive control. mDCs were analyzed for the activation of mDC metabolism and cytokine secretion. The Warburg Effect, glucose concentration in the medium, metabolic rates, and cytokine secretion were determined 72 h post-stimulation **(B)**. Data are mean results of three independent experiments ± SD. Data displayed a gaussian normal distribution. For statistical analysis a ONE-way ANOVA with correction for multiple comparisons according to Tukey was applied. Statistical significance was achieved at *p<0.05, **p<0.01, ***p<0.001, respectively with “n.s.” representing non-significant results.

To further investigate the molecular mechanisms underlying the AIT product 1-induced activation of metabolism and cytokine secretion, mDCs were pre-treated with the SAP/JNK MAPK inhibitor SP600125, then stimulated with AIT product 1, and analyzed for their metabolism and cytokine secretion (**Figure 6A**).

Here, pre-treatment with SP600125 dose-dependently inhibited the AIT product 1-induced Warburg Effect, glucose consumption, and metabolic rates (**Figure 6B**). Moreover, SAP/JNK MAPK-inhibition dose-dependently suppressed the AIT product 1-induced TNF-α- and IL-10 secretion (**Figure 6B**) while dose-dependently increasing IL-1β secretion by approx. 50% (**Figure 6B**). Therefore, these results suggest that the activation of mDC metabolism and cytokine secretion by AIT product 1 depends on both mTOR- and SAP/JNK MAPK-signaling.

### Glutamine- and especially glucose availability is important for the activation of mDCs by AIT product 1

**Figure 7:**
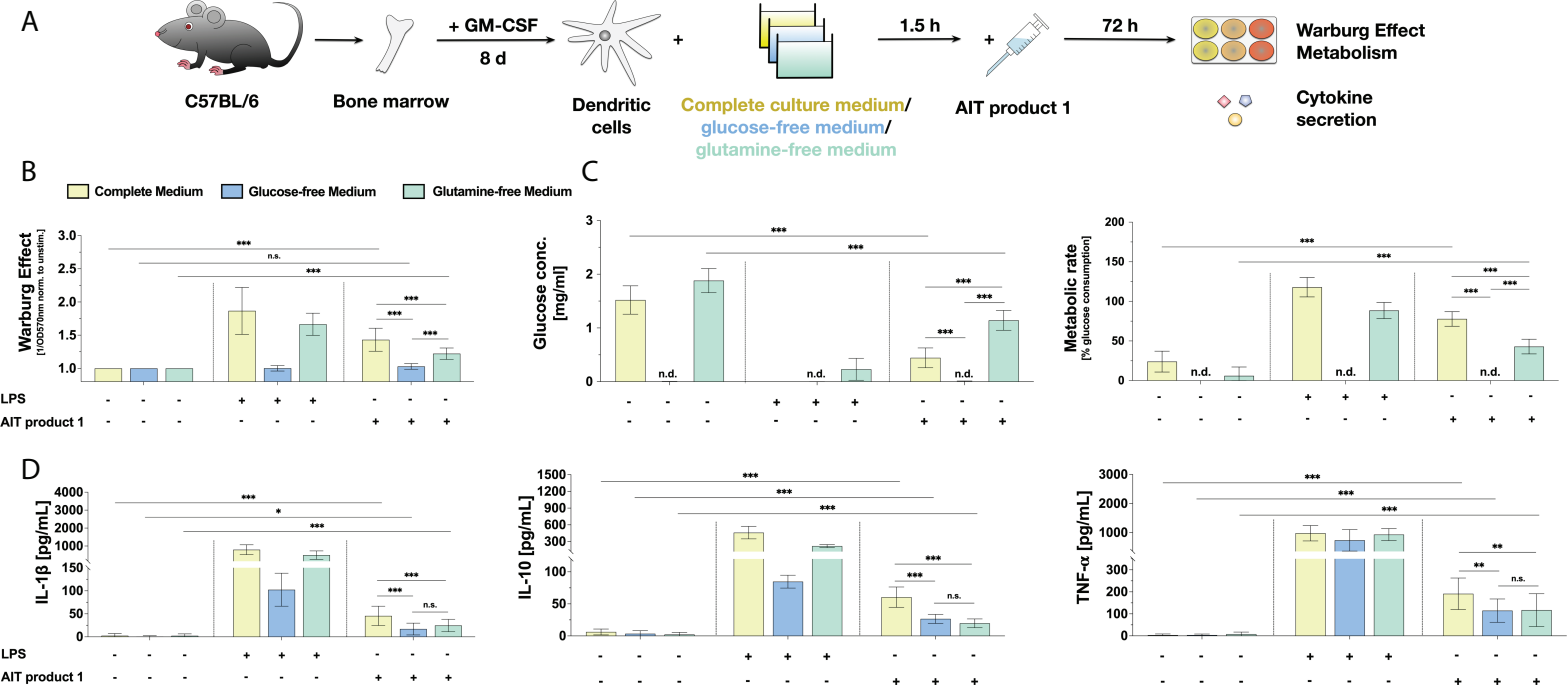
The stimulation of mDCs with AIT product 1 in different culture media demonstrates a glucose- and glutamine-dependency for the activation of mDC metabolism and cytokine secretion. C57BL/6 bone marrow-derived mDCs were stimulated with 2.5 PNU/ml of AIT product 1 (containing 0.38 µg/ml MPLA) for 72 h in either complete culture medium, glucose-free, or glutamine-free medium. 10 µg/ml LPS served as a positive control. mDCs were subsequently analyzed for the activation of mDC metabolism and cytokine secretion **(A)**. The Warburg Effect **(B)**, glucose concentration in the medium and metabolic rates **(C)**, and cytokine secretion **(D)** were determined 72 h post-stimulation. n.d. = not detectable due to lack of glucose in medium. Data are mean results of three independent experiments ± SD. Data displayed a gaussian normal distribution. For statistical analysis a ONE-way ANOVA with correction for multiple comparisons according to Tukey was applied. Statistical significance was achieved at *p<0.05, **p<0.01, ***p<0.001, respectively with “n.s.” representing non-significant results.

To investigate how the availability of the energy sources glutamine and glucose affects their activation, mDCs were stimulated with AIT product 1 in either the previously used complete culture medium, glucose-, or glutamine-free media for 1.5 hours. After the incubation, mDCs were stimulated with AIT product 1 for additional 72 hours in the respective media and analyzed for activation of glucose metabolism and cytokine secretion (**Figure 7A**).

Here, the induced Warburg Effect was significantly lower in AIT product 1-stimulated cells cultured in glutamine-free medium, whereas glucose deprivation completely suppressed the Warburg Effect otherwise observed in complete culture medium (**Figure 7B**). The increases in metabolic rates and the strong consumption of around 70% of available glucose observed in AIT product 1-stimulated mDCs in complete culture medium were also significantly reduced in glutamine-free medium to approx. 40% (**Figure 7C**). As expected, no residual glucose could be detected in glucose-free medium (**Figure 7C**).

The lack of either glucose or glutamine in the culture medium also influenced cytokine secretion: the secretion of IL-1β (reduced by 56% in glucose-free medium compared to complete culture medium vs. 67% reduction in glutamine-free medium compared to complete culture medium), TNF-α (reduction by 40% in glucose-free medium vs. 39% reduction in glutamine-free medium) and IL-10 (reduction by 63% in glucose-free medium vs. 46% reduction in glutamine-free medium) all decreased in the absence of either glucose or glutamine (**Figure 7D**).

In summary, these results suggest that the lack of certain nutrients like glucose or glutamine hinders the AIT product 1-induced activation of mDCs both regarding their metabolic function and cytokine production ability.

### mDC glucose metabolism contributes to the T cell priming capacity of AIT product 1

**Figure 8:**
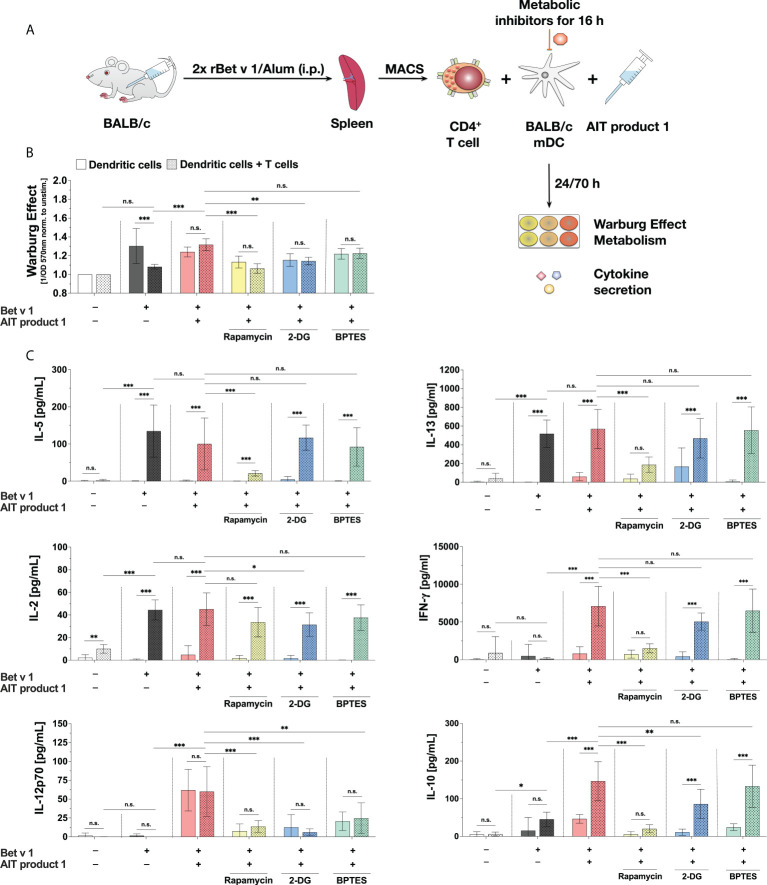
The stimulation of mDC:T cell co-cultures with AIT product 1 leads to increased secretion of Th1 cytokines. BALB/C mDCs were co-cultured with CD4+ T cells isolated from spleens of BALB/C mice that were previously immunized with the major birch pollen allergen Bet v 1 and Alum. Prior to co-cultures, mDCs were pre-treated for 16 hours with metabolic inhibitors (5 nM rapamycin, 0.5 mM 2-DG, or 1 µM BPTES), then the medium was changed, T cells were added, and co-cultures were re-stimulated with either 2 µg/ml Bet v 1 alone or together with 2.5 PNU/ml of AIT product 1 (containing 0.38 µg/ml MPLA) for additional 70 hours **(A)**. The Warburg Effect was determined in mDCs (bars without pattern) and the co-cultures (bars with pattern) **(B)**. Cytokine secretion was determined for IL-2 24 h post-stimulation and for IL-5, IL-13, IFN-γ, IL-12p70, IL-10, IL-6, TNF-α, and IL-1β 72 h post-stimulation **(C)**. Data are mean results of three independent experiments ± SD. Data displayed a gaussian normal distribution. For statistical analysis a ONE-way ANOVA with correction for multiple comparisons according to Tukey was applied. Statistical significance was achieved at *p<0.05, **p<0.01, ***p<0.001, respectively with “n.s.” representing non-significant results.

To determine how the observed activation of mDC metabolism by AIT product 1 contributes to their T cell priming capacity, mDCs were co-cultured with Bet v 1-specific CD4+ T cells. For this, mDCs were pre-treated with the metabolic inhibitors rapamycin, 2-DG, or BPTES for 16 hours, washed, and added to T cells that were freshly isolated from spleens of BALB/c mice immunized twice intraperitoneally (i.p.) against the major birch pollen allergen Bet v 1 using Alum as an adjuvant (**Figure 8A**). The co-cultures were then stimulated with either Bet v 1 alone, or together with AIT product 1. Seventy hours post-stimulation, the Warburg Effect and cytokine secretion were determined (**Figure 8A**).

Stimulation of the co-cultures with Bet v 1 plus AIT product 1 resulted in an increased Warburg Effect (**Figure 8B**). The treatment with both either the glycolysis inhibitor 2-DG or the mTOR inhibitor rapamycin led to significant decreases in the Warburg Effect, whereas BPTES did not have a comparable effect. Additionally, the Warburg Effect did not differ between mDCs alone or the co-cultures neither upon stimulation with Bet v 1 plus AIT product 1 nor for the groups that were pre-treated with all investigated inhibitors (**Figure 8B**).

Additional stimulation of Bet v 1-treated co-cultures with AIT product 1 led to a slight decrease in IL-5 secretion of around 20% while IL-13 secretion was unchanged (**Figure 8C**). For both Th2 cytokines, inhibition of the mTOR pathway *via* rapamycin resulted in a significant decrease in Th2 cytokine secretion from the co-cultures (IL-5: 79% reduction, IL-13: 67% reduction, **Figure 8C**), while pre-treatment of the mDCs with either 2-DG or BPTES had no influence on Bet v 1-induced Th2 cytokine secretion (**Figure 8C**).

T cell activation by Bet v 1 resulted in a strong increase in IL-2 secretion from the co-cultures in comparison to the unstimulated controls which was only slightly and non-significantly increased by the addition of AIT product 1 (**Figure 8C**). Pre-treatment with the inhibitors rapamycin, 2-DG, and BPTES slightly decreased IL-2 secretion (by 21%, 30%, and 17%, respectively after 24 hours, **Figure 8C**).

AIT product 1 stimulation also significantly increased the secretion of IL-1β (20 pg/ml in rBet v 1 re-stimulated co-cultures vs. 380 pg/ml in co-cultures additionally stimulated with AIT product 1, **Repository Figure E5B**), TNF-α (2 pg/ml vs. 45 pg/ml, **Repository Figure E5B**), IL-10 (46 pg/ml vs. 147 pg/ml), IL-12p70 (0 pg/ml vs. 60 pg/ml), and IFN-γ (120 pg/ml vs. 7097 pg/ml) from the co-cultures compared to the controls re-stimulated with rBet v 1 alone (**Figure 8C**). Here, the secretion of IFN-γ, IL-12p70, IL-10, and IL-1β was found to be significantly decreased upon inhibition of both mTOR-signaling by pre-treatment with rapamycin and, to a lesser extent, hexokinase 2 inhibition by 2-DG (**Figure 8C** and **Repository Figure E5**). The amino acid metabolism inhibitor BPTES did not affect the secretion of any cytokine in BALB/c-derived cells, except for a decrease in AIT product 1-induced IL-12p70 secretion (by approx. 60%) in both stimulations of mDCs alone as well as mDC:TC co-cultures (**Figure 8C**)

In summary, our results showed mTOR-dependent glucose metabolism in mDC to be critical for the Th1-biased T cell priming capacity of AIT product 1-stimulated mDCs.

## Discussion

In the present study, we compared the allergen therapeutic Pollinex^®^ Quattro (termed AIT product 1), containing the TLR4-ligand and clinically used adjuvant MPLA, to four Alum-adjuvanted AIT products as well as to two commercial, MPLA-adjuvanted vaccines. In our experimental system, both the MPLA-containing AIT product 1 and two tested commercial vaccines, but not the Alum-adjuvanted AIT products, modulated the induction of immune responses by both changing the metabolic state of mDCs and inducing a pronounced cytokine secretion.

Interestingly, except for a slight upregulation of all investigated surface markers by AIT product 5, slightly increased expression levels of CD40, CD80, and CD86 upon stimulation by AIT product 3, and a weak upregulation of CD86 by AIT product 2 and 4, the allergoid preparations adjuvanted with Alum did neither activate mDC metabolism nor induce TNF-α secretion. In line with our results, Benito‐Villalvilla et al. described Alum to inhibit mTOR-activation in human DCs that were stimulated by allergoids conjugated to nonoxidized-mannan (27). The resulting inhibition of Warburg metabolism by Alum reduced both production of IL-10 and expression of PD-L1, thereby impairing the capacity of the human DCs to generate functional regulatory T cells while at the same time promoting mixed Th1-/Th2-/Th17-responses (27). These results likely explain why the Alum-adjuvanted allergen preparations tested in this study lacked the capacity to activate Warburg metabolism in mDCs.

Human tolerogenic DCs were reported to display both an enhanced glycolytic capacity and ROS production compared to mature pro‐inflammatory DCs (28). Therefore, a better understanding of the glycolytic metabolism induced by MPLA is of high interest in scenarios where the induction of regulatory DC phenotypes is considered beneficial (e.g. in the treatment of allergies or autoimmunity).

For our studies, we decided to rely on bone marrow-derived, GM-CSF-differentiated myeloid dendritic cells, termed mDCs. GM-CSF-differentiated bone marrow cultures contain both DCs and macrophages (29). To minimize contaminating macrophages in our mDC preparations, we only harvested the loosely adherent cells on day 8 of culture without scratching the strongly adherent macrophages, resulting in only ~1.4% F4/80+ cells in our CD11b+CD11c+populations (data not shown).

Here, both C57BL/6 and BALB/c-derived mDC preparations showed similar responses to the investigated stimuli (albeit weaker for BALB/c-derived mDCs), while C57BL/6-derived Flt-3L-differentiated cultures containing plasmacytoid dendritic cells (pDCs) did not respond to either the AIT products or the commercial vaccines.

Upon activation, the GM-CSF-derived mDC preparations used throughout our study, similarly to M1 macrophages, mainly rely on mTOR-dependent glycolytic metabolism (30). For pDCs, the contribution of different metabolic pathways to the immune responses initiated and controlled by these cells is less clear. While mTOR is involved in the TLR9-induced type I interferon signaling pathway (23), and stimuli such as the TLR7-agonist gardiquimod or Influenza induce early glycolysis in pDCs (31), the effect of TLR4-ligands on the metabolism of pDCs is not well studied.

Moreover, pDC functions were reported to be also strongly modulated by both cholesterol- and fatty acid-metabolism (reviewed in (32)). In contrast, we did not observe a significant contribution of fatty acid metabolism to the AIT product 1-induced immune responses in our experiments in mDCs. Therefore, our results as well as the available literature suggest mDCs and pDCs to be metabolically distinct cell types which have elegantly adapted to their respective niches and main effector functions.

Using the MPLA-adjuvanted AIT product 1 as a model, we analyzed the induced alterations in DC metabolism, the underlying mechanisms, and their contribution to the effector responses of mDCs in more detail.

While the Th1-promoting effects of AIT product 1 *in vivo* in pre-clinical models and in patients are well described (11, 33, 34), so far, sufficient clinical human data fully supporting marketing authorization were not reported. Moreover, to our knowledge, the effects of AIT product 1 on DCs are unknown. In our experiments, AIT product 1 dose-dependently activated mDCs inducing both pro- (IL-1β and TNF-α) and anti-inflammatory (IL-10) cytokine secretion. Furthermore, AIT product 1 was able to more strongly induce the expression of CD40, CD69, CD80, and CD86 compared to all Alum-adjuvanted AIT products. This mDC activation was paralleled by a pronounced increase in glucose metabolism, as evidenced by the induced Warburg Effect and the increased glucose consumption from the culture medium. AIT product 1 induced both pro- and anti-inflammatory cytokine secretion from mDCs. However, the results of the DC:TC co-culture experiments showed the overall immune responses to be pro-inflammatory, with the mDCs secreting high amounts of IL-1β and TNF-α and the T cells producing IL-2 and IFN-γ while at the same time maintaining the Bet v 1-induced secretion of the Th2 cytokines IL-5 and IL-13.

Besides tree pollen allergens from birch, alder, and hazel (19), AIT product 1 contains microcrystalline L-Tyrosine used as a depot and MPLA as an adjuvant to boost immune responses (17). To understand which of these components mediate the observed mDC activation, mDCs were stimulated with either formulations of AIT product 1 containing different allergen amounts or the single adjuvant components contained within AIT product 1. We observed no differences in mDC activation between the AIT product 1 formulations that contained different allergen amounts. We recently reported the TLR4-ligand MPLA to activate glucose metabolism in mDCs (18). In line with these results, our experiments showed the immune-metabolic effects of AIT product 1 to be primarily mediated by the contained MPLA as stimulation with corresponding amounts of MPLA alone (but not L-Tyrosine) resulted in an activation of mDC metabolism and cytokine secretion that was comparable to the complete AIT product 1 formulation.

Moreover, pre-treatment of the mDCs with either the mTOR-inhibitor rapamycin or the SAP/JNK MAPK inhibitor SP600125) dose-dependently inhibited the induced Warburg Effect, glucose consumption, and metabolic rates. mTOR- and SAP/JNK MAPK-inhibition moreover dose-dependently suppressed AIT product 1-induced TNF-α and IL-10 secretion while SAP/JNK MAPK-inhibition increased IL-1β secretion. Therefore, these results suggest the activation of mDC glucose metabolism and anti-inflammatory cytokine secretion by AIT product 1 to depend on both mTOR- and SAP/JNK MAPK-signaling, while pro-inflammatory cytokine secretion was at least in part mediated by SAP/JNK MAPK-signaling. As expected, these results are in accordance with our recent results showing the activation of mDCs by MPLA to also depend on both mTOR- and MAPK-signaling (18).

We previously showed that, compared to LPS, MPLA-stimulation induced similar but attenuated immune responses in several important immune cell types such as mouse epithelial cells, mDCs, B-, and T cells, as well as human ex vivo-isolated monocytes, while being unable to activate either human or mouse mast cells (13). In line with these results, a recent study compared LPS- and MPLA mediated activation of human blood cells on a transcriptional level and found that LPS and MPLA share upstream regulators and have comparable effects on canonical pathways and cellular functions (35). While several pro-inflammatory cytokines (e.g. IL-6, TNF-α, or IL-1A) were more strongly induced by LPS, the macrophage-regulating chemokine CCL7 was the only factor found to be preferentially upregulated by MPLA (35).

As the results obtained after mTOR-inhibition suggested mDC-activation by AIT product 1 to depend on mDC metabolism, we performed additional experiments in which we either inhibited mTOR-signaling, glucose-, amino acid-, or fatty acid metabolism in AIT product 1-stimulated mDCs by pre-treatment with different inhibitors. Here, AIT product 1-induced mDC activation was strongly reduced upon inhibition of mTOR-signaling (suppression of Warburg Effect, glucose consumption, IL-10, and TNF-α secretion). Moreover, inhibition of the glycolytic enzyme hexokinase 2 by 2-DG and amino acid metabolism by BPTES reduced AIT product 1-induced mDC activation. In contrast, fatty acid metabolism seemed to be dispensable for AIT product 1-mediated mDC activation. These results were confirmed by the cultivation of AIT product 1-stimulated mDCs in media that lacked either glucose or glutamine. Here, both glucose- and glutamine deprivation strongly reduced the AIT product 1-induced Warburg Effect and cytokine secretion.

Inside the cell, glutamine is also used for the generation of uridine diphosphate (UDP)-N-acetyl-glucosamine (GlcNAc), which is an essential substrate for subsequent glycosylation reactions (36). Since the rate-limiting enzyme for the generation of UDP-GlcNAc, glutamine:fructose-6-phosphate aminotransferase (GFAT) catalyzes the conversion of fructose-6-phosphate to glucosamine-6-phosphate with glutamine donating its amino group to become glutamate, glutamine is crucial to ensure proper protein function as well as signal transduction through glycosylation reactions (36, 37). It could already be shown for macrophages that stimulation with LPS leads to an enhanced expression of GFAT affecting protein glycosylation (38).

In contrast to complete glucose deprivation, the glutaminase inhibitor BPTES only blocks the conversion of glutamine to glutamate within the mitochondria (24). Therefore, the dependency of protein glycosylation on glutamine might explain why the deprivation of glutamine from the culture medium affected both the glycolytic shift as well as the cytokine secretion in our experimental setup.

To our knowledge, we are the first to report the immune-metabolic effects of commercial AIT products in mDCs. In line with our results, Fensterheim et al. reported the adjuvant MPLA to trigger a MyD88-, TRIF-, and mTOR-dependent metabolic switch towards Warburg metabolism in macrophages associated with augmented phagocytosis and respiratory burst that supported improved pathogen clearance *in vivo* and *in vitro* (39). In the reported study, early TLR4-driven aerobic glycolysis in macrophages was coupled with late mitochondrial biogenesis, enhanced malate shuttling, and increased mitochondrial ATP production (39). This metabolic reprogramming likely allows MPLA-primed macrophages to both sustain mitochondrial ATP production and use glycolytic- and mitochondrial byproducts for antimicrobial purposes (40–42).

Finally, to investigate how the observed metabolic changes in AIT product 1-stimulated mDCs contributed to their T cell priming capacity, we performed co-cultures of AIT product 1-stimulated mDCs with Bet v 1-specific CD4+ T cells. Interestingly, while the suppression of Bet v 1-induced Th2 cytokine secretion by AIT product 1 was minimal, we observed a pronounced induction of the Th1 cytokine IFN-γ as well as the mDC-derived, Th1-promoting cytokines TNF-α and IL-12p70 in co-cultures stimulated with AIT product 1. These results are in accordance with the described capacity of AIT product 1 to induce mainly Th1-biased immune responses and reduce allergic symptoms *in vivo* both in animal models and in humans (11, 33, 34). *In vitro* co-application of MPLA with grass pollen allergen was reported to result in an IL-12-dependent reduction of IL-5 secretion from PBMCs while up-regulating IFN-γ production (43). Moreover, human monocytes and monocyte-derived DCs stimulated with MPLA displayed enhanced allergen uptake, DC maturation, and the promotion of IFN-γ producing Th1 cells, albeit without induction of T cell-derived IL-10 (44). In our experimental system, inhibition of mTOR-signaling in mDCs massively disrupted the capacity of the mDCs to produce the cytokines IL-10 and IL-12p70 and to induce both Th1- and Th2-responses in allergen-specific T cells. Therefore, our results clearly showed the activation of mTOR-dependent mDC metabolism to be essential for the induction of adaptive immune responses.

While we thoroughly analyzed the cytokine responses of both mDCs and T cells in the co-culture setting, one weakness of this study is that we did not address the activation status of the T cells in terms of cell surface-expressed activation markers. Moreover, while the effects on the activation, metabolism, and *in vitro* Th1-priming capacity of mDCs by AIT product 1 were investigated by us in detail, their contribution to the reported Th1-priming capacity of AIT product 1 *in vivo* needs further investigation and remains a task for future studies.

In summary, our work demonstrated that both an AIT product and two commercial vaccines adjuvanted with MPLA could activate mDC metabolism. As a model, we thoroughly characterized the contribution of mDC metabolism to the responses of these important cells to the allergen therapeutic AIT product 1. We found that the activation of mDCs by AIT product 1 is mediated by a pronounced mTOR- and SAP/JNK MAPK-dependent activation of glucose metabolism that regulates mDC-derived cytokine secretion. mDC glucose metabolism was also critical for the (Th1-biased) T cell priming capacity of AIT product 1-stimulated mDCs.

With our work, we are the first to investigate both the activation of mDCs by AIT product 1 and to describe its immune-metabolic effects. Further investigating the contribution of immune metabolism to the overall immune responses induced by both certain types of immune cells, different stimuli, or the used adjuvants will help us to both better understand and improve vaccination and AIT.

## Data availability statement

The raw data supporting the conclusions of this article will be made available by the authors, without undue reservation.

## Ethics statement

The animal study was reviewed and approved by RP Darmstadt.

## Author contributions

JZ, CM, and AG: Data curation, formal analysis, investigation, visualization, writing – review & editing; SWe and SWo: Formal analysis, investigation; Y-JL: Formal analysis, investigation, writing – review & editing; GS: Resources, writing – review & editing; FF: Conceptualization, methodology, resources, writing – review & editing; SV: Conceptualization, resources, writing – review & editing; SSche: Conceptualization, funding acquisition, writing – review & editing; SSchu: Funding acquisition, conceptualization, data curation, project administration, supervision, visualization, writing – original draft. All authors contributed to the article and approved the submitted version.

## Funding

This work was in part funded by the budget of the Paul-Ehrlich-Institut, Langen, Germany. AG was funded by the German Research Foundation (DFG SCHU2951/4). Y-JL was funded by the German Research Foundation (DFG SCHE637/4).

## Conflict of interest

The authors are employees of the German Federal Institute for Vaccines and Biomedicines. The Paul-Ehrlich-Institut (PEI) is an Agency of the German Federal Ministry of Health. In relation to the present publication, the authors consider themselves not having a conflict of interest. Opinions expressed in the paper are personal views of the authors, not necessarily reflecting an official opinion of the PEI or the German Federal Ministry of Health.

## Publisher’s note

All claims expressed in this article are solely those of the authors and do not necessarily represent those of their affiliated organizations, or those of the publisher, the editors and the reviewers. Any product that may be evaluated in this article, or claim that may be made by its manufacturer, is not guaranteed or endorsed by the publisher.

## Supplementary material

Supplementary Figure 1 An MPLA-containing AIT product can activate the metabolism of BALB/c-derived myeloid dendritic cells. BALB/c bone marrow-derived mDCs were stimulated with the different AIT products and vaccines (all corresponding to 5 PNU/ml of AIT product 1, see for coding and composition of the products and for protein concentrations), or 10 µg/ml LPS as a positive control for 72 h and analyzed for the activation of mDC metabolism and cytokine secretion. The Warburg Effect, glucose consumption from the culture medium, and TNF-α secretion were determined 72 h post-stimulation. Data are mean results of three independent experiments ± SD. Statistical comparisons were performed in relation to AIT product 1-stimulated samples. Data displayed a gaussian normal distribution. For statistical analysis a ONE-way ANOVA with correction for multiple comparisons according to Tukey was applied. Statistical significance was achieved at *p<0.05, **:p<0.01, ***:p<0.001, respectively with “n.s.” representing non-significant results.

Supplementary Figure 2 C57BL/6-derived plasmacytoid dendritic cells are not activated by either AIT products of MPLA-containing vaccines. C57BL/6 bone marrow-derived pDCs were stimulated with the different AIT products and vaccines (all corresponding to 5 PNU/ml of AIT product 1, see for coding and composition of the products and for protein concentrations), or 10 µg/ml LPS as a positive control for 72 h and analyzed for the activation of pDC metabolism and cytokine secretion. The Warburg Effect, glucose consumption from the culture medium, and TNF-α secretion were determined 72 h post-stimulation. Data are mean results of three independent experiments ± SD. Statistical comparisons were performed in relation to AIT product 1-stimulated samples. Data displayed a gaussian normal distribution. For statistical analysis a ONE-way ANOVA with correction for multiple comparisons according to Tukey was applied. Statistical significance was achieved at *:p<0.05, **:p<0.01, ***:p<0.001, respectively, with “n.s.” representing non-significant results.

Supplementary Figure 3 The stimulation of mDCs with AIT product 1 activates mDC metabolism and cytokine secretion. C57BL/6 bone marrow-derived mDCs were stimulated with either the indicated increasing concentrations of AIT product 1 or 10 µg/ml LPS as a positive control for 72 h and analyzed for the activation of mDC metabolism and cytokine secretion. The Warburg Effect, glucose concentration in the medium, metabolic rates, and cytokine secretion were determined 72 h post-stimulation **(A)**. For Extracellular Flux Assays, mDCs adhered to Seahorse Assay Plates were stimulated with a concentration of 15 PNU/ml AIT product 1 (containing 2 µg/ml of MPLA) for 14 cycles (84 min) with or without pre-treatment with the hexokinase 2 inhibitor 2-DG (50 mM) for 8 cycles (48 min) and analyzed for extracellular acidification rates (ECAR) and oxygen consumption rates (OCR) using Seahorse technology **(B)**. Data are mean results of either three independent experiments ± SD (A) or representative results from three independent experiments normalized to total protein content **(B)**. Data displayed a gaussian normal distribution. For statistical analysis a ONE-way ANOVA with correction for multiple comparisons according to Tukey was applied. Statistical significance was achieved at *:p<0.05, **:p<0.01, ***:p<0.001, respectively with “n.s.” representing non-significant results.

Supplementary Figure 4 The activation of mDC metabolism and cytokine secretion by AIT product 1 is independent of allergen content. C57BL/6 bone marrow-derived mDCs were stimulated with either increasing amounts (0.076 µl/ml, 0.76 µl/ml, 7.6 µl/ml, or 15.2 µl/ml, containing either 0.0038, 0.038, 0.38, or 0.76 µg/ml of MPLA, respectively) of different formulations of AIT product 1 containing the indicated allergen concentrations or 10 µg/ml LPS as a positive control for 72h and analyzed for the activation of mDC metabolism and cytokine secretion **(A)**. The Warburg Effect **(B)** and the secretion of the indicated cytokines **(C-E)** were determined 72 h post-stimulation. Data are mean results of three independent experiments ± SD. Data displayed a gaussian normal distribution. For statistical analysis a ONE-way ANOVA with correction for multiple comparisons according to Tukey was applied. Statistical significance was achieved at *:p<0.05, **:p<0.01, ***:p<0.001, respectively with “n.s.” representing non-significant results.

Supplementary Figure 5 The stimulation of mDC: T cell co-cultures with AIT product 1 leads to increased secretion of IL-1β and TNF-α. BALB/C mDCs were co-cultured with CD4+ T cells isolated from spleens of BALB/C mice that were previously immunized with the major birch pollen allergen Bet v 1 and Alum. Prior to co-cultures, mDCs were pre-treated for 16 hours with metabolic inhibitors (5 nM rapamycin, 0.5 mM 2-DG, 1 µM BPTES), then medium was changed, T cells were added, and co-cultures were re-stimulated with either 2 µg/ml Bet v 1 alone or together with 2.5 PNU/ml of AIT product 1 (containing 0.38 µg/ml MPLA) for additional 70 hours **(A)**. IL-1β and TNF-α secretion was determined by ELISA **(B)**. Data are mean results of three independent experiments ± SD. Data displayed a gaussian normal distribution. For statistical analysis a ONE-way ANOVA with correction for multiple comparisons according to Tukey was applied. For statistical analyses, a two-way ANOVA with correction for multiple comparisons according to Tukey was performed. Statistical significance was achieved at *:p<0.05, **:p<0.01, ***:p<0.001, respectively with “n.s.” representing non-significant results.

## Abbreviations

2-DG, 2-deoxyglucose; Alum, aluminum salt; AS, adjuvant system; AUM, arbitrary unit; BEH, birch, elder, hazel; BPTES, bis-2-(5-phenylacetamido- 1,3,4-thiadiazol-2-yl)-ethyl-sulfide; ECAR, extracellular acidification rate; Flt-3L, FMS-like tyrosine kinase 3 ligand; GFAT, glutamine:fructose-6-phosphate; GlcNAc, N-acetyl-glucosamine aminotransferase; GM-CSF, granulocyte-macrophage colony-stimulating factor; LPS, lipopolysaccharide; mDC, myeloid dendritic cell; MAPK, mitogen-activated protein kinase; MPLA, monophosphoryl lipid A; mTOR, mammalian target of rapamycin; mTORC1, mTOR complex 1; NOS, reactive nitrogen species; OCR, oxygen consumption rate; PAMP, pathogen-associated molecular pattern; pDC, plasmacytoid dendritic cell; PNU, protein nitrogen unit; rFlaA:Betv1, fusion protein consisting of the TLR5-ligand flagellin and the major birch pollen allergen Bet v 1; ROS, reactive oxygen species; SAP/JNK, stress-activated protein kinases/Jun amino-terminal kinase; SU, standardized unit; TC, T cell; TE, therapeutic units; TNF-α, tumor necrosis factor alpha; TLR, “Toll”-like receptor; UDP, uridine diphosphate.

## Glossary

**Table:**

2-DG	2-deoxyglucose
Alum	aluminum salt
AS	adjuvant system
AUM	arbitrary unit
BEH	birch
elder	hazel
BPTES	bis-2-(5-phenylacetamido- 1,3,4-thiadiazol-2-yl)-ethyl-sulfide
ECAR	extracellular acidification rate
Flt-3L	FMS-like tyrosine kinase 3 ligand
GFAT	glutamine:fructose-6-phosphate
GlcNAc	N-acetyl-glucosamine aminotransferase
GM-CSF	granulocyte-macrophage colony-stimulating factor
LPS	lipopolysaccharide
mDC	myeloid dendritic cell
MAPK	mitogen-activated protein kinase
MPLA	monophosphoryl lipid A
mTOR	mammalian target of rapamycin
mTORC1	mTOR complex 1
NOS	reactive nitrogen species
OCR	oxygen consumption rate
PAMP	pathogen associated molecular pattern
pDC	plasmacytoid dendritic cell
PNU	protein nitrogen unit
rFlaA:Betv1	fusion protein consisting of the TLR5-ligand flagellin and the major birch pollen allergen Bet v 1
ROS	reactive oxygen species
SAP/JNK	stress-activated protein kinases/Jun aminoterminal kinase
SU	standardized unit
TC	T cell
TE	therapeutic units;
TNF-a	tumor necrosis factor alpha
TLR	“Toll”-like receptor
UDP	uridine diphosphate.
