# Supplementary figures and images for: Modulation of dendritic cell metabolism by an MPLA-adjuvanted allergen product for specific immunotherapy
Source: Front Immunol. 2022 Aug 17;13:916491. doi: 10.3389/fimmu.2022.916491 (PMC9430023; doi:10.3389/fimmu.2022.916491)

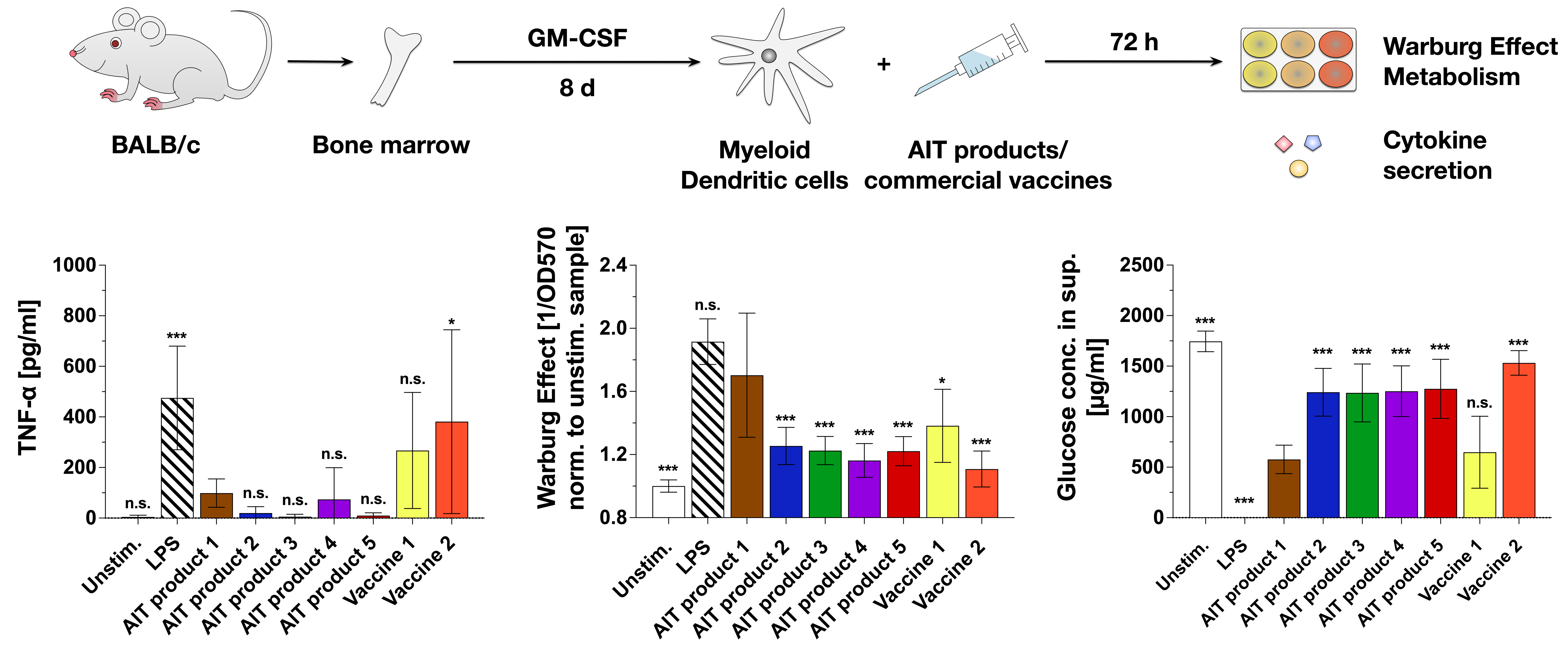

Supplement: Supplementary Figure 1 — An MPLA-containing AIT product can activate the metabolism of BALB/c-derived myeloid dendritic cells. BALB/c bone marrow-derived mDCs were stimulated with the different AIT products and vaccines (all corresponding to 5 PNU/ml of AIT product 1, see for coding and composition of the products and for protein concentrations), or 10 µg/ml LPS as a positive control for 72 h and analyzed for the activation of mDC metabolism and cytokine secretion. The Warburg Effect, glucose consumption from the culture medium, and TNF-α secretion were determined 72 h post-stimulation. Data are mean results of three independent experiments ± SD. Statistical comparisons were performed in relation to AIT product 1-stimulated samples. Data displayed a gaussian normal distribution. For statistical analysis a ONE-way ANOVA with correction for multiple comparisons according to Tukey was applied. Statistical significance was achieved at *p<0.05, **:p<0.01, ***:p<0.001, respectively with “n.s.” representing non-significant results. [file Image_1.tiff]

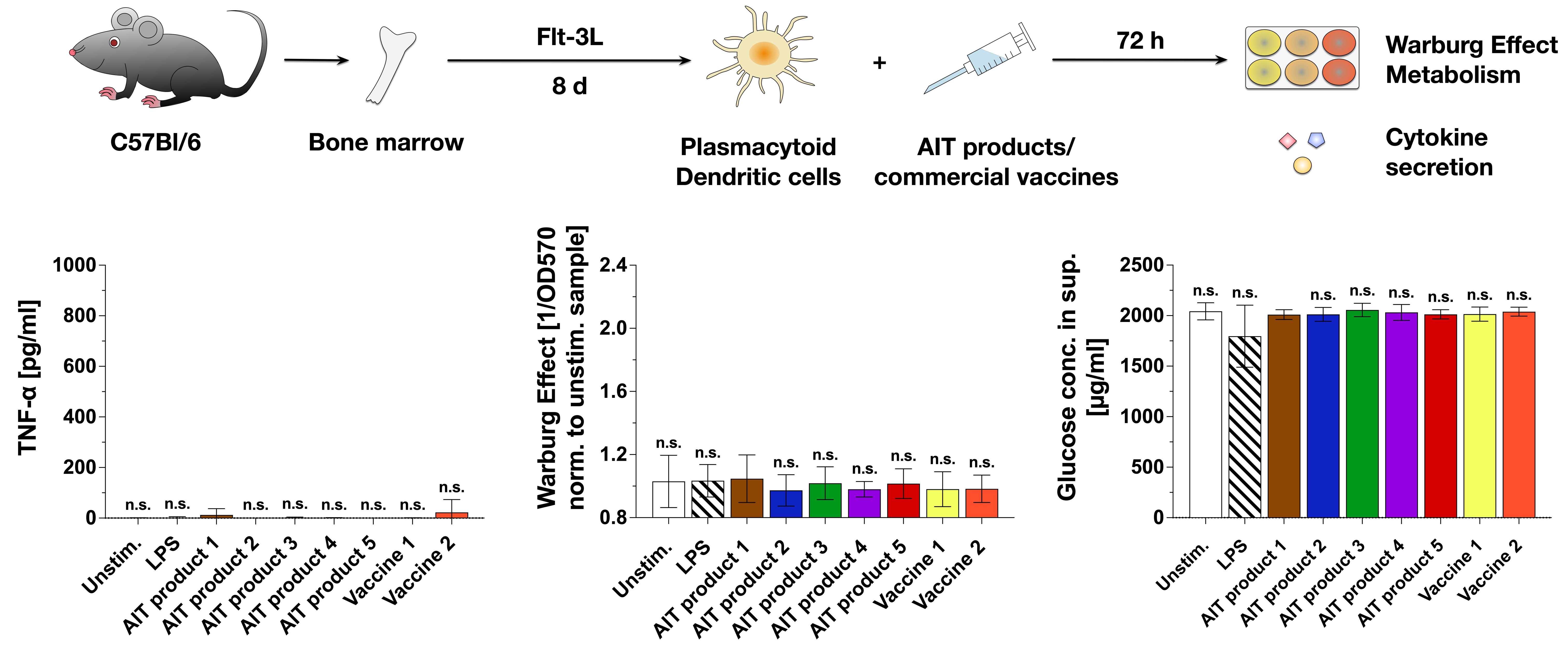

Supplement: Supplementary Figure 2 — C57BL/6-derived plasmacytoid dendritic cells are not activated by either AIT products of MPLA-containing vaccines. C57BL/6 bone marrow-derived pDCs were stimulated with the different AIT products and vaccines (all corresponding to 5 PNU/ml of AIT product 1, see for coding and composition of the products and for protein concentrations), or 10 µg/ml LPS as a positive control for 72 h and analyzed for the activation of pDC metabolism and cytokine secretion. The Warburg Effect, glucose consumption from the culture medium, and TNF-α secretion were determined 72 h post-stimulation. Data are mean results of three independent experiments ± SD. Statistical comparisons were performed in relation to AIT product 1-stimulated samples. Data displayed a gaussian normal distribution. For statistical analysis a ONE-way ANOVA with correction for multiple comparisons according to Tukey was applied. Statistical significance was achieved at *:p<0.05, **:p<0.01, ***:p<0.001, respectively, with “n.s.” representing non-significant results. [file Image_2.tiff]

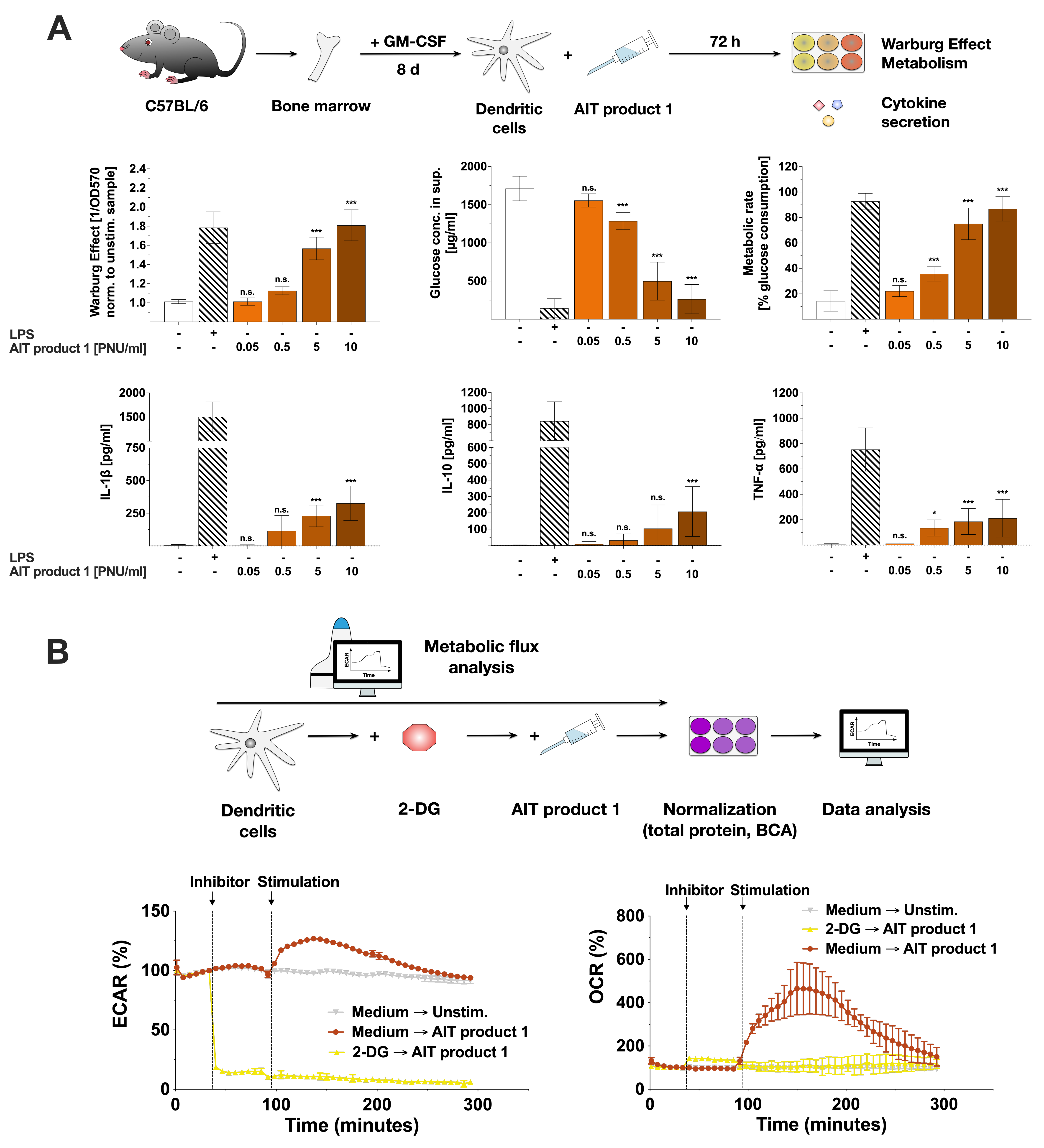

Supplement: Supplementary Figure 3 — The stimulation of mDCs with AIT product 1 activates mDC metabolism and cytokine secretion. C57BL/6 bone marrow-derived mDCs were stimulated with either the indicated increasing concentrations of AIT product 1 or 10 µg/ml LPS as a positive control for 72 h and analyzed for the activation of mDC metabolism and cytokine secretion. The Warburg Effect, glucose concentration in the medium, metabolic rates, and cytokine secretion were determined 72 h post-stimulation (A). For Extracellular Flux Assays, mDCs adhered to Seahorse Assay Plates were stimulated with a concentration of 15 PNU/ml AIT product 1 (containing 2 µg/ml of MPLA) for 14 cycles (84 min) with or without pre-treatment with the hexokinase 2 inhibitor 2-DG (50 mM) for 8 cycles (48 min) and analyzed for extracellular acidification rates (ECAR) and oxygen consumption rates (OCR) using Seahorse technology (B). Data are mean results of either three independent experiments ± SD (A) or representative results from three independent experiments normalized to total protein content (B). Data displayed a gaussian normal distribution. For statistical analysis a ONE-way ANOVA with correction for multiple comparisons according to Tukey was applied. Statistical significance was achieved at *:p<0.05, **:p<0.01, ***:p<0.001, respectively with “n.s.” representing non-significant results. [file Image_3.tiff]

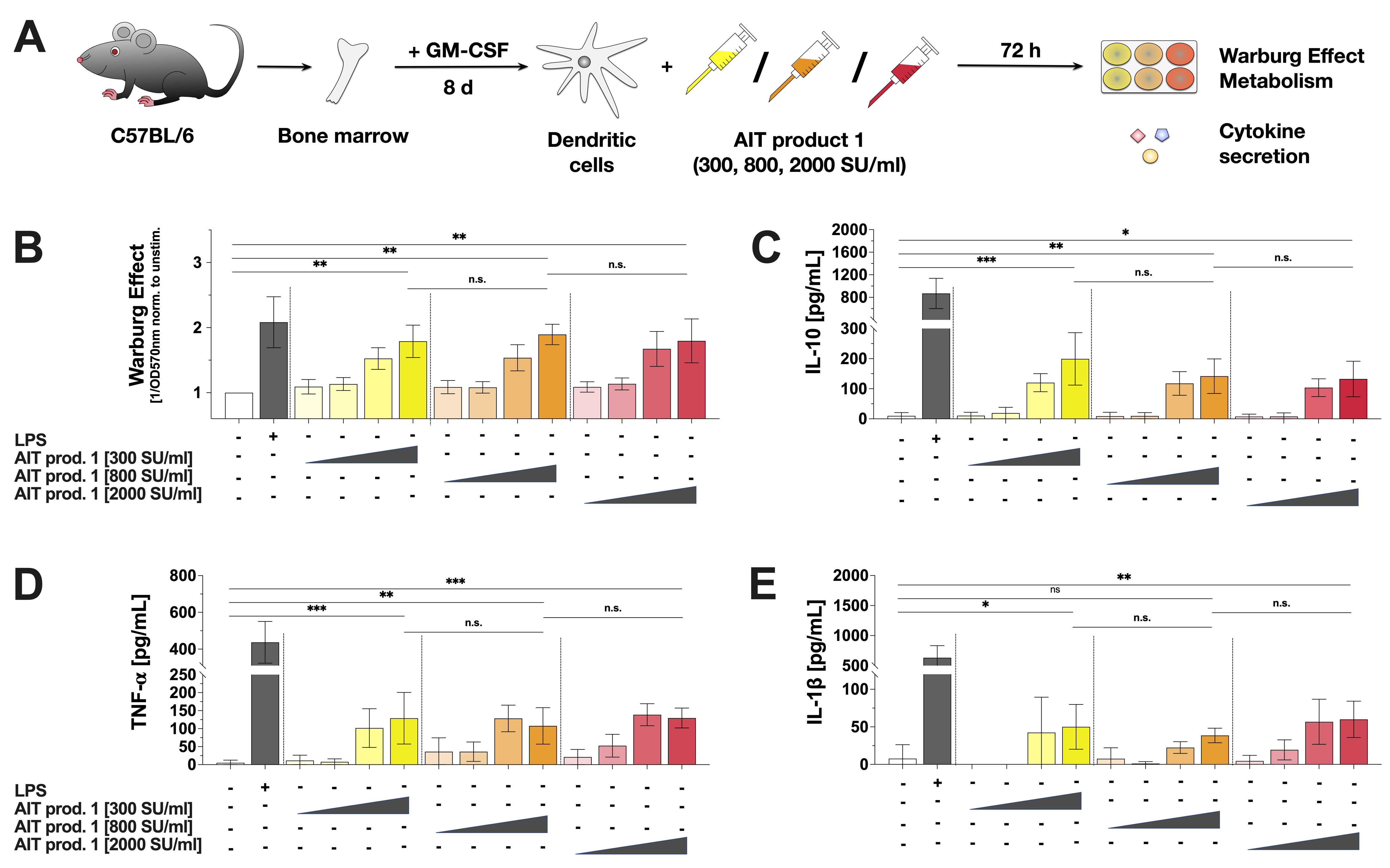

Supplement: Supplementary Figure 4 — The activation of mDC metabolism and cytokine secretion by AIT product 1 is independent of allergen content. C57BL/6 bone marrow-derived mDCs were stimulated with either increasing amounts (0.076 µl/ml, 0.76 µl/ml, 7.6 µl/ml, or 15.2 µl/ml, containing either 0.0038, 0.038, 0.38, or 0.76 µg/ml of MPLA, respectively) of different formulations of AIT product 1 containing the indicated allergen concentrations or 10 µg/ml LPS as a positive control for 72h and analyzed for the activation of mDC metabolism and cytokine secretion (A). The Warburg Effect (B) and the secretion of the indicated cytokines (C-E) were determined 72 h post-stimulation. Data are mean results of three independent experiments ± SD. Data displayed a gaussian normal distribution. For statistical analysis a ONE-way ANOVA with correction for multiple comparisons according to Tukey was applied. Statistical significance was achieved at *:p<0.05, **:p<0.01, ***:p<0.001, respectively with “n.s.” representing non-significant results. [file Image_4.tiff]

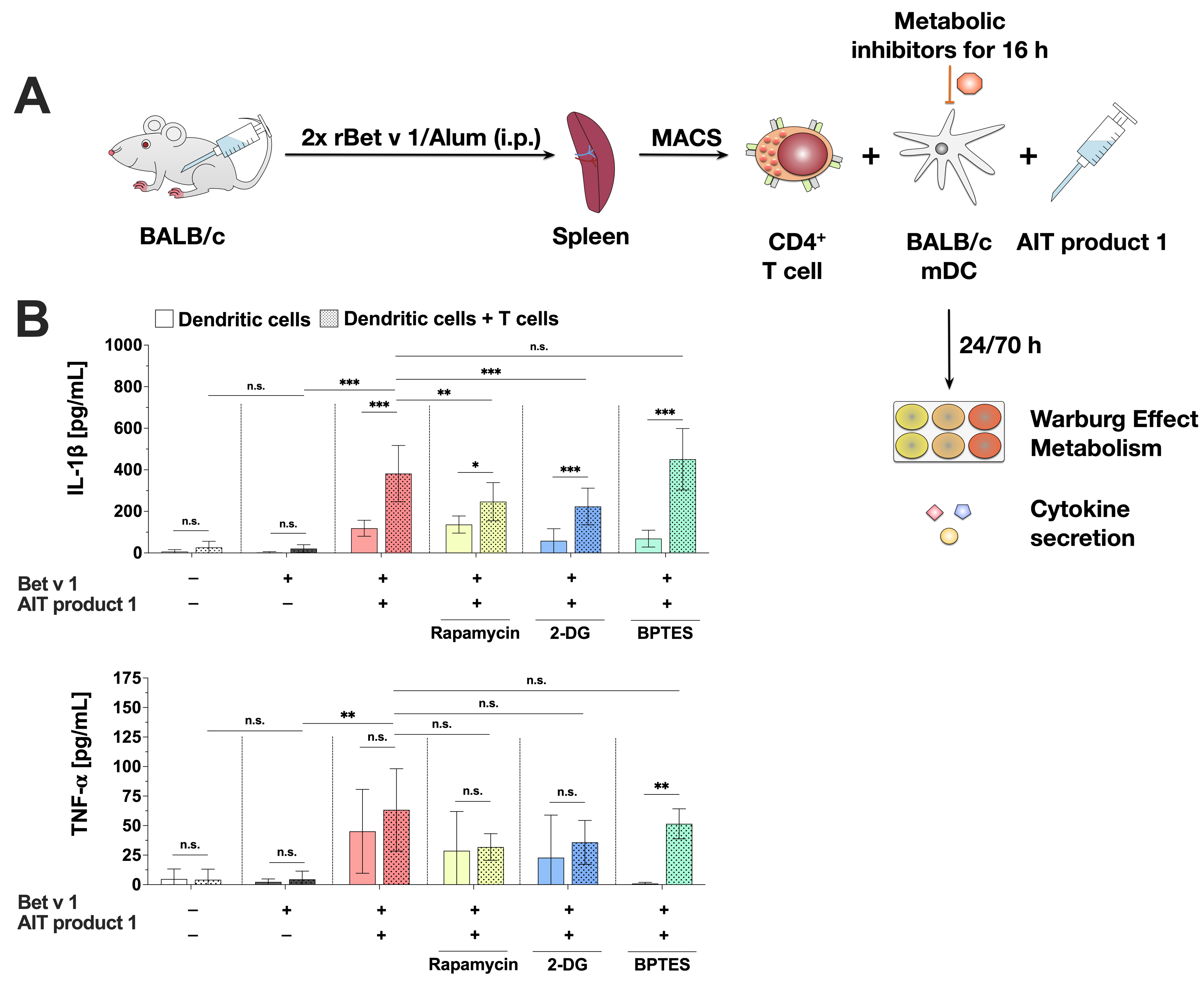

Supplement: Supplementary Figure 5 — The stimulation of mDC: T cell co-cultures with AIT product 1 leads to increased secretion of IL-1β and TNF-α. BALB/C mDCs were co-cultured with CD4+ T cells isolated from spleens of BALB/C mice that were previously immunized with the major birch pollen allergen Bet v 1 and Alum. Prior to co-cultures, mDCs were pre-treated for 16 hours with metabolic inhibitors (5 nM rapamycin, 0.5 mM 2-DG, 1 µM BPTES), then medium was changed, T cells were added, and co-cultures were re-stimulated with either 2 µg/ml Bet v 1 alone or together with 2.5 PNU/ml of AIT product 1 (containing 0.38 µg/ml MPLA) for additional 70 hours (A). IL-1β and TNF-α secretion was determined by ELISA (B). Data are mean results of three independent experiments ± SD. Data displayed a gaussian normal distribution. For statistical analysis a ONE-way ANOVA with correction for multiple comparisons according to Tukey was applied. For statistical analyses, a two-way ANOVA with correction for multiple comparisons according to Tukey was performed. Statistical significance was achieved at *:p<0.05, **:p<0.01, ***:p<0.001, respectively with “n.s.” representing non-significant results. [file Image_5.tiff]
